# Tuning the Cell-Adhesive Properties of Two-Component Hybrid Hydrogels to Modulate Cancer Cell Behavior, Metastasis, and Death Pathways

**DOI:** 10.1021/acs.biomac.2c00733

**Published:** 2022-09-22

**Authors:** Melis Isik, Babatunde O. Okesola, Cemil Can Eylem, Engin Kocak, Emirhan Nemutlu, Emel Emregul, Matteo D’Este, Burak Derkus

**Affiliations:** † Interdisciplinary Research Unit for Advanced Materials (INTRAM), Department of Chemistry, Faculty of Science, Ankara University, Ankara 06560, Turkey; ‡ Department of Eye and Vision Science, Institute of Life Course and Medical Sciences, Faculty of Medicine, University of Liverpool, Liverpool L7 8TX, U.K.; § School of Life Science, Faculty of Medicine and Health Sciences, University of Nottingham, Nottingham NG7 2RD, U.K.; ∥ Analytical Chemistry Division, Faculty of Pharmacy, Hacettepe University, Ankara 06230, Turkey; ⊥ Division of Analytical Chemistry, Faculty of Gulhane Pharmacy, Health Science University, Ankara 06018, Turkey; # Bioanalytic and Omics Laboratory, Faculty of Pharmacy, Hacettepe University, Ankara 06230, Turkey; ∇ AO Research Institute Davos, Clavadelerstrasse 8, Davos Platz 7270, Switzerland; ¶ Stem Cell Research Lab, Department of Chemistry, Faculty of Science, Ankara University, Ankara 06560, Turkey

## Abstract

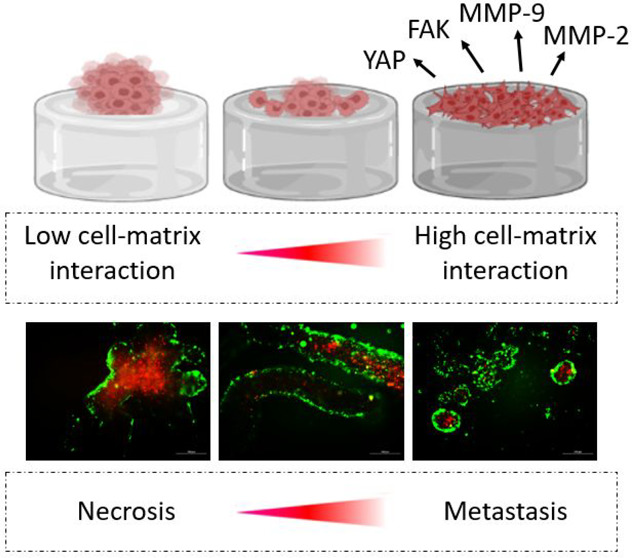

This work presents a polysaccharide and protein-based two-component hybrid hydrogel integrating the cell-adhesive gelatin-tyramine (G-Tyr) and nonadhesive hyaluronic acid-tyramine (HA-Tyr) through enzyme-mediated oxidative coupling reaction. The resulting HA-Tyr/G-Tyr hydrogel reflects the precise chemical and mechanical features of the cancer extracellular matrix and is able to tune cancer cell adhesion upon switching the component ratio. The cells form quasi-spheroids on HA-Tyr rich hydrogels, while they tend to form an invasive monolayer culture on G-Tyr rich hydrogels. The metastatic genotype of colorectal adenocarcinoma cells (HT-29) increases on G-Tyr rich hydrogels which is driven by the material’s adhesive property, and additionally confirmed by the suppressed gene expressions of apoptosis and autophagy. On the other hand, HA-Tyr rich hydrogels lead the cells to necrotic death via oxidative stress in quasi-spheroids. This work demonstrates the ideality of HA-Tyr/G-Tyr to modulate cancer cell adhesion, which also has potential in preventing primary metastasis after onco-surgery, biomaterials-based cancer research, and drug testing.

## 1 Introduction

The biophysical and biochemical properties of the tumor microenvironment play critical roles in controlling cancer etiology and progression.^1^ Tumors grow in extracellular matrixes (ECMs) composed of proteins, glycoproteins, proteoglycans, and polysaccharides.^2,3^ These ECM components present a spectrum of biophysical properties (stiffness, permeability, composition, spatial organization, and topography) and bioactive cues that play key roles in regulating cancer cell behaviors including cell–cell interactions, metastasis, invasion, apoptosis, and resistance to therapy.^4,5^ In addition, the fibrillary network of collagen, fibrin, or fibronectin in the native ECM provides specific cell adhesion receptors and promotes the formation of spatially controlled tumor capsules that guide interactions between cancer cells and stroma cells. The proteolytic degradation of ECM components can lead to progressive destruction of the normal ECM and its replacement with a tumor-derived ECM in a time-dependent fashion.^6^ The dynamic ECM remodeling and the reciprocal effects on cell–ECM and cell–cell interactions in the complex tumor microenvironment underpin the transition of cancer cells from an epithelial phenotype to an aggressive mesenchymal phenotype (Epithelial-Mesenchymal Transition, EMT), high invasive capability, and resistance to chemotherapeutics.^7,8^ Given the interplay of the biophysical and biochemical properties of the tumor ECM on the pathophysiological mechanisms of various types of cancer, there is an increasing need to recreate the tumor microenvironment that recapitulates critical mechanical and biochemical cues present in the natural ECM while facilitating cancer cell adhesion, aggregation, migration, and apoptosis *in vitro*.

Hydrogel scaffolds are attractive synthetic analogs of the native ECM due to their ability to provide a spectrum of relevant biophysical properties. Hydrogels possess high water content, facile transport of nutrients, soluble growth factors, oxygen, and waste. Furthermore, hydrogels can be designed under mild and biocompatible conditions to have a tunable structure, morphology, shape, stiffness, and topography and present cell adhesion ligands.^9^ Hydrogel scaffolds have emerged as a promising biomimetic platform to model fundamental cancer cell biology.^10^ Hydrogels that can modulate various stages of metastasis can provide insight into the cause-effect cycle of cancer. Most studies that aim to probe the effects of ECM biophysical and biochemical properties on cancer metastasis rely on the synergistic effects of multiple material properties that hydrogels scaffolds provide.^11,12^ Hydrogel stiffness is an important element commonly harnessed to simulate how ECM biophysical property regulate cancer cell phenotypic and genotypic fates *in vivo*.^13^

Both natural and synthetic materials have been used to create biomimetic hydrogel scaffolds to elucidate the mechanisms underpinning metastasis and invasiveness in cancer cells. Notably, hydrogel scaffolds created with proteins such as collagen,^14^ Matrigel,^15^ or fibrin^16^ are extensively used to mimic tumor microenvironment for cancer cell culture and drug screening due to their specific biophysical and cell-adhesive properties as well as their important roles in tumor progression, invasion, and metastasis. However, protein-based hydrogels are limited by poor mechanical properties. On the other hand, polysaccharide and synthetic polymer-based hydrogels formed with hyaluronic acid (HA),^17^ agarose,^18^ alginate,^19^ or polyethylene glycol derivatives^20,21^ have useful biophysical properties to model tumor microenvironment. However, they lack cell-adhesive epitopes and thus, require additional chemical modification with, for example, arginylglycylaspartic acid (RGD) peptides to incorporate adhesion sites.^22^

The possibility to integrate the mechanical property of polysaccharides or polymers and the cell-adhesive properties of proteins in hydrogel design can facilitate the creation of new material scaffolds with tunable mechanical properties, diversity in morphology, tunable cell–matrix interactions, and multiple biological activities. Multicomponent hydrogels composed of polysaccharides and proteins offer a broad range of biophysical and biochemical properties to replicate tumor microenvironment and control cancer cell fate. Two-component hydrogels created with alginate and Matrigel have been designed to enable independent tuning of matrix stiffness and composition to control the morphology and malignant cell phenotype in mammary epithelium.^23^ Three-component hydrogels composed of alginate, collagen and agarose support the growth of multicellular cancer spheroids from human ovarian cancer cells.^24^ Similarly, hybrid hydrogels created with a decellularized tissue and cellulose displayed the capability to upregulate the expression of aggressive cancer related genes.^25^ Taking advantage of the unique tunable biophysical property and surface chemistry of multicomponent hydrogels, the possibility to modulate cell–cell and matrix-cell interactions that occur on the 2D surface of a 3D hydrogel presents a unique opportunity to simulate and control cancer cell behaviors along the surface of a tissue basement membrane.

In this work, we designed two-component functional hydrogels integrating the cell-adhesive property of gelatin (G) and nonadhesive property of hyaluronic acid. Both G and HA were conjugated with tyramine (G-Tyr and HA-Tyr), that brings unique advantages such as fast (<1 min) hydrogel formation through enzyme-mediated oxidative coupling reaction. By tuning the ratio of gelatin to hyaluronic acid in the hydrogels, we demonstrated the possibility to modulate the mechanical property, cell–cell, cell–matrix interactions, and molecular phenotype using colorectal adenocarcinoma cells (HT-29) as an exemplar. Furthermore, the possibility to tune the surface chemistry of the two-component hydrogels enabled us to control cell adhesion and switch between cell monolayer formation and cell clustering (sphere formation) on the 2D surface of 3D hydrogels. HT-29 cell line cultured on 2D surface of 3D gelatin-hyaluronic acid hydrogels showed significant alterations in the cancer cell metastasis and death pathway related (apoptosis, autophagy, and necrosis)-related genes and the associated metabolite profiles.

## 2 Experimental Section

### 2.1 Cell Culture

HT-29 cells were cultured in Dulbecco’s Modified Eagle’s Medium-High Glucose (DMEM-HG, Thermo Fisher, USA) supplemented with fetal bovine serum (FBS, 10% v/v, Thermo Fisher, USA) and penicillin-streptomycin (P/S, 1% v/v, Thermo Fisher, USA) in a CO_2_ incubator at 37 °C and 5% CO_2_ conditions. The medium was refreshed every 3 days until the cells reached the confluence.

### 2.2 Preparation of Hybrid HA-Tyr/G-Tyr Hydrogels

Enzymatically cross-linkable G-Tyr was synthesized by a condensation reaction between gelatin and tyramine hydrochloride through *N*-hydroxysulfosuccinimide (NHS)/1-Ethyl-3-(3-(dimethylamino)propyl) carbodiimide hydrochloride (EDC) chemistry (detailed synthesis and characterization available in the Supporting Information (SI)). Pure HA-Tyr (synthesized and characterized previously)^26^ and G-Tyr powders were dissolved in Dulbecco’s Phosphate Buffer Saline (DPBS) at desired concentrations (1% and 3% wt HA-Tyr; 5% and 10% wt G-Tyr). Horse radish peroxidase (HRP, 2 U/mL) was added to the prepared gel solutions for oxidative coupling. To prepare hybrid hydrogels with varying HA-Tyr and G-Tyr ratios, G-Tyr (5% or 10% wt) and HA-Tyr (1% and 3% wt) were mixed at the ratios of 2:1, 1:1, and 1:2 (v/v). Hydrogelation of HA-Tyr, G-Tyr, and hybrid gels was triggered by addition of hydrogen peroxide (H_2_O_2_, 2 mM) and incubation at room temperature for 1 min.

### 2.3 Characterization of Hybrid HA-Tyr/G-Tyr Hydrogels

#### Chemical Characterization

For the structural characterization of HA-Tyr/G-Tyr hybrid hydrogels, we first performed ATR-FTIR (Shimadzu-Infinity FTIR spectrometer, Japan) analysis for HA-Tyr, G-Tyr, and HA-Tyr/G-Tyr to confirm the presence of specific functional groups in HA-Tyr/G-Tyr hybrid hydrogels. The spectra were recorded in the range of 4000–500 cm^–1^ with a 2 cm^–1^ spectral resolution. Additional notes can be found in the SI.

#### Micromechanical Characterization

Briefly, G-Tyr (5 and 10% wt), HA-Tyr (1 and 3% wt), and HA-Tyr/G-Tyr (1–10% wt) (2:1, 1:1, 1:2, v/v) hydrogels were prepared in cylindrical molds with an 8 mm diameter and a 1 cm height. After complete gelation upon enzymatic cross-linking with HRP/H_2_O_2_, the gels were placed on the lower plate of the testing instrument (CellScale, Canada) with a capacity of 10 kN load and were compressed at a speed of 0.1 mm/min at room temperature. The compressive force was recorded until the hydrogels were deformed. A stress–strain curve was plotted during the measurement. The compressive modulus of each sample was assessed as a ratio of the stress and strain in the linear area of the stress–strain curve.

#### Micro-nanoscale Characterization

The microstructures of hybrid gel components, namely, HA-Tyr and G-Tyr as well as optimized hybrid hydrogel HA-Tyr/G-Tyr (1–10% wt), at three different volumetric ratios (2:1, 1:1, 1:2 v/v) were investigated via scanning electron microscopy (SEM, FEI 430 Nova NanoSEM, USA). The samples after freeze-drying were sputter-coated with gold (Quorom SC7640 High Resolution Sputter Coater, Lewes, UK), and the images were acquired at 10–15 kV.

#### Liquid Displacement Test

The porosity (%) of hybrid hydrogels prepared in volumetric ratios of 2:1, 1:1, and 1:2 (v/v) was determined by the liquid displacement method.^27^ The detailed experimental procedure is presented in the SI.

### 2.4 Cell Viability and Proliferation on the Hydrogel Components

To examine the potential cytotoxic effects of gel components on cell viability and proliferation, live/dead staining and XTT tests were performed for synthesized G-Tyr (5% and 10% wt) as well as previously obtained HA-Tyr (1% and 3% wt) hydrogels prior to proceeding with further experiments. To visualize cell survival on HA-Tyr (1 and 3% wt) and G-Tyr (5 and 10% wt), HT-29 cells (40 000 cell/gel) were seeded on hydrogels and cultured for up to 7 days, and then the cells were stained with calcein-AM (4 μM, Molecular Probes, Thermo Fisher, UK) to monitor live cells and with ethidium homodimer-1 (EthD-1, 2 μM, Molecular Probes, Thermo Fisher, UK) to monitor the dead cells. The cells were observed under a fluorescent microscope (Leica DMIL, Germany) at 488 nm (green) and 527 nm (red) wavelengths. To further analyze the cell proliferation, we also performed an XTT test (Biological Industries, USA). Following a culture period of HT-29 cells seeded atop HA-Tyr (1 and 3% wt) and G-Tyr (5 and 10% wt) hydrogels for up to 7 days, the cells were washed and treated with XTT reagent (50 μL). Following 3 h of incubation, Absorbance values were recorded at 490 nm with a microplate spectrophotometer (Multiskan Sky, Thermo Fisher, USA). Each quantitative experiment was performed with three replicates. The statistical difference between more than two groups was investigated by one-way ANOVA followed by Tukey’s posthoc test.

### 2.5 Optimization of Hybrid HA-Tyr/G-Tyr Hydrogels to Control Cell Adhesion

To visualize the effects of HA-Tyr/G-Tyr hybrid hydrogels on cancer cell adhesion, HT-29 cells were seeded on hybrid hydrogels prepared with varying component concentrations (1% HA-Tyr-5% G-Tyr, 1% HA-Tyr-10% G-Tyr, 3% HA-Tyr-5% G-Tyr, and 3% HA-Tyr-10% G-Tyr). In addition, different combination ratios (2:1, 1:1, 1:2 v/v) of HA-Tyr to G-Tyr were tested. After 2 and 7 days of incubation, the cells (40 000 cell/gel) were stained with calcein-AM as previously described. The capacity of the cells to form a monolayer culture or cell clusters/aggregates was assessed under a fluorescent microscope at 488 nm wavelength (green).

### 2.6 Assessing Cell Adhesion through Immunostaining for β-Actin and YAP

To visualize the cell adhesion and cell–matrix interaction, we performed an immunofluorescent staining for cell cytoskeleton protein (β-actin) and mechanosensing protein Yes-associated protein (YAP). After 5-days of culture of HT-29 cells (40 000 cells/gel) on the varying ratios (2:1, 1:1, 1:2 v/v) of hybrid hydrogels (HA-Tyr(1%)- G-Tyr(10%)), the cells were fixed with paraformaldehyde (PFA, 4% v/v, Sigma) for 15 min followed by treating with Triton X-100 (0.1% v/v, Sigma) for ensuring the permeability. Nonspecific binding was prevented by an incubation step with bovine serum albumin (BSA, 0.1% v/v, Sigma), which was followed by inserting the primary antibodies β-actin (1:200 v/v, Santa Cruz, USA) or YAP (1:200 v/v, Santa Cruz, USA). After an overnight incubation at 4 °C, the gels were washed and labeled with secondary anti-mouse antibody conjugated with Alexa Fluor 488 (1:1000 v/v, Thermo Fisher, USA). Nuclear staining was performed with DAPI (Thermo Fisher, USA). immunofluorescence images were obtained with a fluorescent microscope (Leica DMIL, Germany) at green and blue channels.

### 2.7 Gene Expression Study

The effect of hybrid HA-Tyr/G-Tyr hydrogels with different component ratios on mechanotransduction (YAP, FAK), EMT (ECad, NCad), metastasis (MMP-2, MMP-9), apoptosis (Casp-3, p53), autophagy (ATG-5, Beclin-1), and necrosis (RIPK1, RIPK3) markers was investigated by reverse transcriptase-quantitative polymerase chain reaction (RT-qPCR). For this purpose, HT-29 cells (400 000 cell/gel) were cultured atop HA-Tyr/G-Tyr (1%–10% wt) hybrid hydrogels at 1:1, 1:2, and 2:1 (v/v) component ratios. After 5 days of culture, total RNA was isolated (GeneDireX, USA) and converted into cDNA using a kit (Bio-Rad, USA). RT-qPCR was run on a thermal cycler (Bio-Rad CFX96 instrument, USA). Primer sequences (Oligomer Biotechnology Jsc., Turkey) are provided in the SI. Each quantitative experiment was performed with three replicates. The statistical difference between more than two groups was investigated by one-way ANOVA followed by Tukey’s posthoc test.

### 2.8 Apoptosis/Necrosis Assay

To visualize the dual effect of HA-Tyr/G-Tyr hybrid hydrogels on apoptosis and necrosis of HT-29 cells, the cells (100 000 cell/gel) were seeded atop HA-Tyr/G-Tyr (1%–10% wt) hybrid hydrogels at 1:1, 1:2, and 2:1 (v/v) component ratios and cultured for 5 days. For the apoptosis-necrosis assay, cells were washed for two times with PBS, and Apopxin Green (stains apoptotic cells) and 7-aminoactinomycin-D (7-AAD) (stains necrotic cells) were applied to the cells to stain apoptotic and necrotic cells. The nuclei were stained with DAPI. After the samples were incubated for 30 min at room temperature, the cells were visualized under a fluorescent microscope at green, red, and blue channels.

### 2.9 Metabolomics Study

Metabolomics study was conducted by gas chromatography–mass spectrometry (GC-MS) and liquid chromatography quadrupole time-of-flight mass spectrometry (LC-qTOF-MS) as we previously reported.^28,29^ Detailed protocols explaining the sample processing, method, data processing, bioinformatics and statistical analysis are provided in the SI.

## 3 Results

### 3.1 Rationale of Design

**Figure 1:**
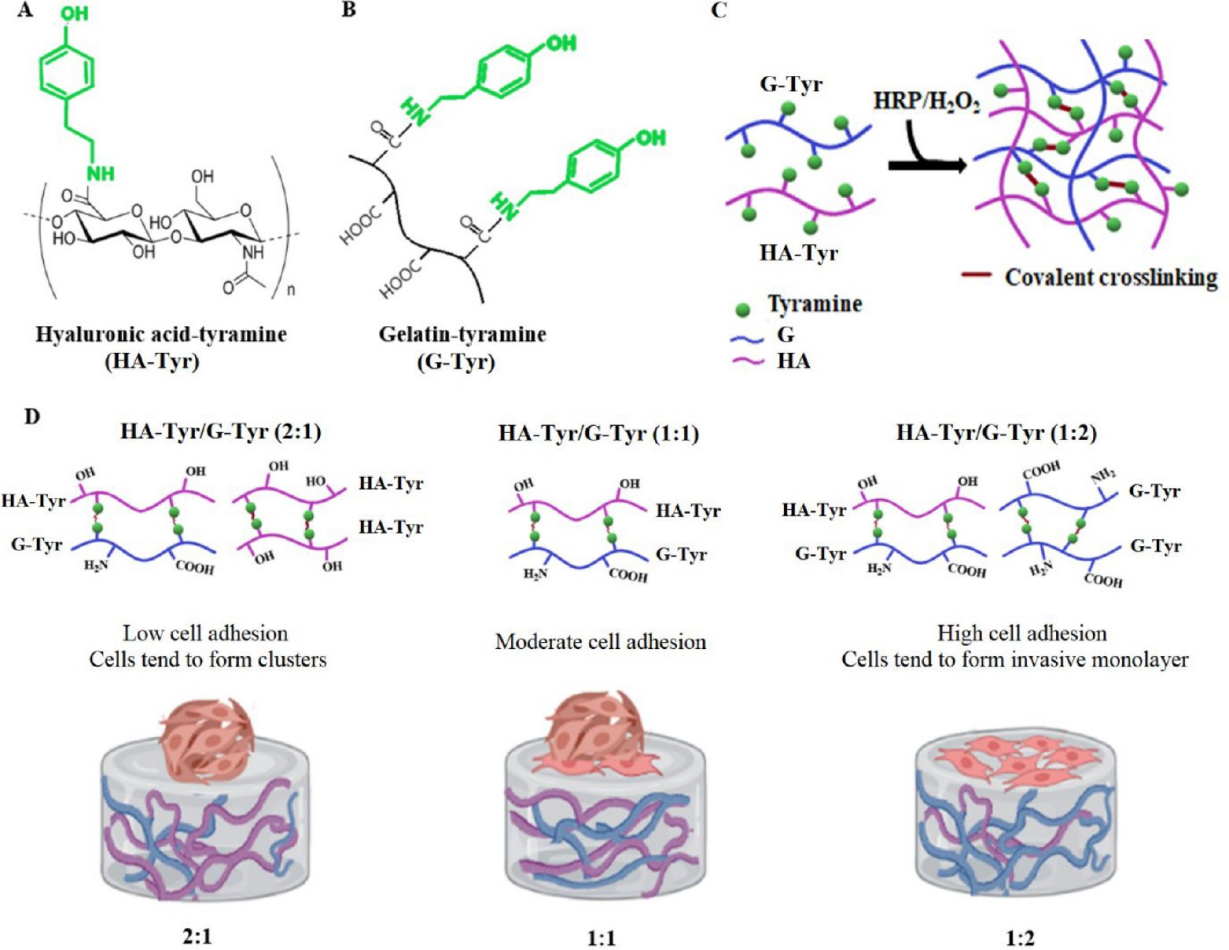
Rationale of design. The components HA-Tyr (A) and G-Tyr (B) form two-component hybrid hydrogels (HA-Tyr/G-Tyr) through a HRP-mediated oxidative coupling reaction (C). Hydrogels with varying fractions are obtained by switching the volumetric ratio of HA-Tyr (noncell-adhering component) to G-Tyr (cell-adhering component) (D). Emerging two-component hydrogels have the ability to tune adhesion of cancer cells and enable switching between cell monolayer formation and cell clustering.

Hyaluronic acid hydrogels have previously been used to induce cell sphere formation while gelatin hydrogel scaffolds are well-known to promote cell adhesion.^30,31^ To enable facile synthesis of the two-component hydrogels, first, we functionalized the carboxylic acid groups of both gelatin and hyaluronic acid with tyramine to produce G-Tyr and HA-Tyr as previously described elsewhere^32,33^ (detailed synthesis are presented in the SI, Figures S1–S8). Then, we used HRP-mediated oxidative coupling to facilitate instant hydrogelation of G-Tyr, HA-Tyr and their combinations in the presence of hydrogen peroxide as we have previously reported^31,34^ (Figure 1A–C). We reasoned that two-component hydrogels formed by combining various ratios of hyaluronic acid and gelatin would present a tunable surface chemistry (integrin-binding surface or nonadhesive surface or mixture) that can modulate the spatial organization of cancer cells at the cell–matrix interface. For example, we anticipate that a two-component hydrogel containing a high concentration of G-Tyr will promote tumor cell adhesion while a hydrogel containing a high concentration of nonadhesive HA-Tyr induces cell clustering on the surface of the hydrogels (Figure 1D), thus mimicking cancer cell–ECM interaction the *in vivo* tumor microenvironment. In this study, our approach relies on the integration of the cell-adhesive property of gelatin and nonadhesive property of hyaluronic acid in a tunable two-component hydrogel with high chemical and mechanical precision to control cancer cell attachment and clustering.

### 3.2 Synthesis and Characterization of Two-Component Hydrogels Based on HA-Tyr and G-Tyr

To assess the ability to form hydrogels in their own right, we prepared aqueous solutions of HA-Tyr at 1 and 3% wt and G-Tyr at 5 and 10% wt gelator concentrations in PBS containing HRP (1% wt). Then we added H_2_O_2_ (5 mM) to the gelator solutions to induce instant hydrogelation (within 20 s) at room temperature. HA-Tyr formed self-supporting but weak hydrogels at 1% wt, whereas it formed robust hydrogels at 3% wt Similarly, G-Tyr formed stable hydrogels at 10% wt but weak hydrogels at 5% wt We also prepared two-component hydrogels with various mixing ratios of HA-Tyr (1 or 3% wt) and G-Tyr (5 or 10% wt). Stable hydrogels were formed with HA-Tyr (1% wt)-G-Tyr (10% wt), HA-Tyr (3% wt)-G-Tyr (10% wt), HA-Tyr (1% wt)-G-Tyr (5% wt), and HA-Tyr (3% wt)-G-Tyr (5% wt), albeit with different mechanical stability.

**Figure 2:**
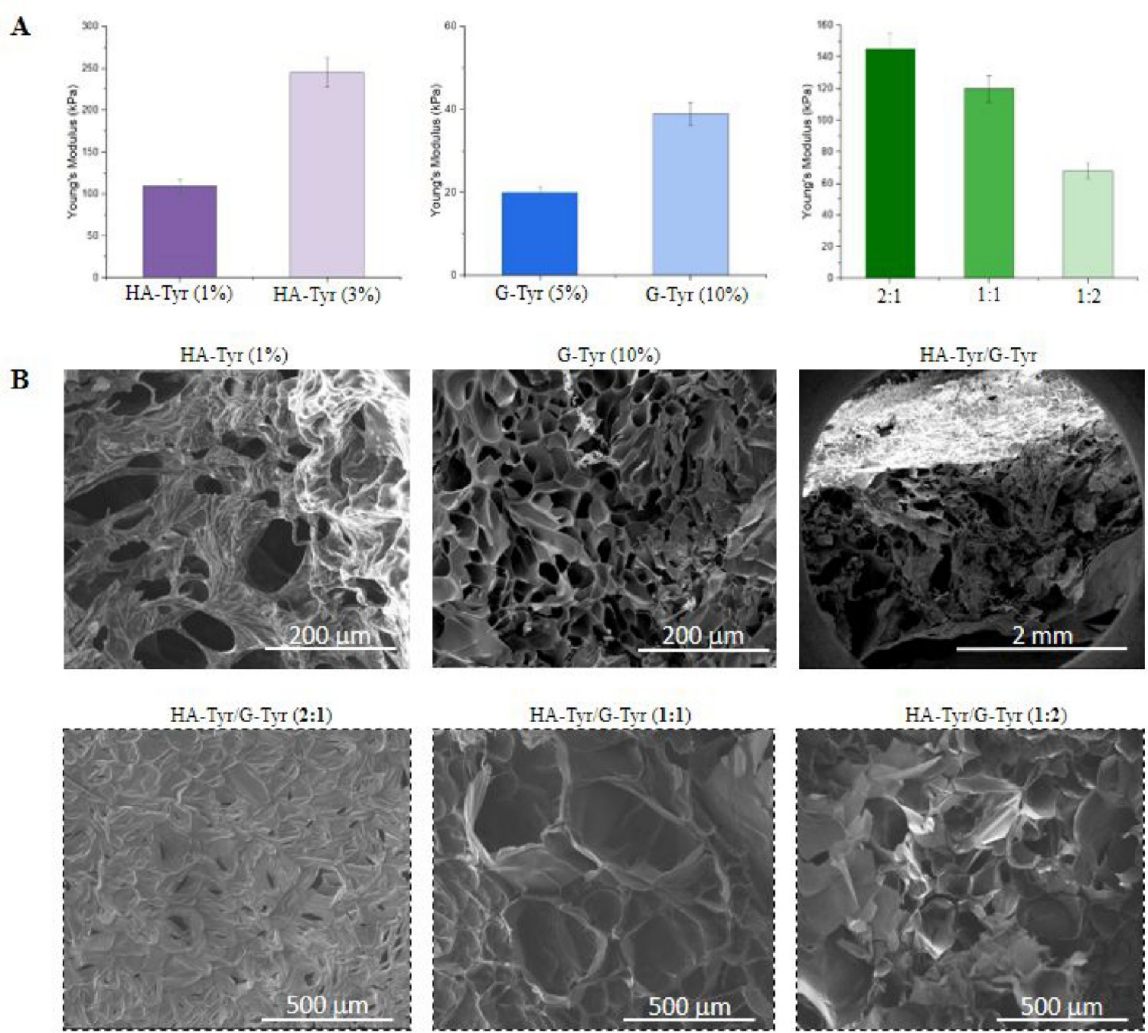
Characterization of HA-Tyr/G-Tyr hybrid hydrogels. (A) Young’s moduli related to HA-Tyr (1 and 3% wt), G-Tyr (1 and 10% wt), and different volumetric ratios (2:1, 1:1, 1:2 v/v) of HA-Tyr/G-Tyr (1%–10% wt). (B) SEM micrographs showing the microstructures of HA-Tyr (1% wt), G-Tyr (10 wt), and HA-Tyr/G-Tyr (1%–10% wt) hybrid hydrogels and of HA-Tyr/G-Tyr (1%–10% wt) hydrogels prepared with varying HA-Tyr to G-Tyr ratio (2:1, 1:1, 1:2 v/v).

To characterize the mechanical properties of the hydrogels, we carried out micromechanical compression testing on the hydrogels (CellScale, Canada). The Young’s modulus values of HA-Tyr at 1 and 3% were found to be approximately 110 and 245 kPa, respectively, while Young’s moduli of G-Tyr at 5 and 10% were found to be 20 and 39 kPa, respectively (Figure 2A). The Young’s moduli of HA-Tyr/G-Tyr (1–10% wt) prepared by mixing HA-Tyr to G-Tyr in 2:1, 1:1, 1:2 volumetric ratios were found to be 143, 120, and 72 kPa, respectively (Figure 2A). Higher compression moduli of hybrid hydrogels (HA-Tyr to G-Tyr = 1:2 to 2:1) showed that the weak mechanical strength of G-Tyr was suppressed in the hybrid HA-Tyr/G-Tyr hydrogel with the increase of HA-Tyr ratio in addition to increasing interactions between HA-Tyr/G-Tyr, which confirms that the mechanical strength can be tuned by changing the HA-Tyr ratio.

The microstructures of HA-Tyr, G-Tyr , and HA-Tyr/G-Tyr hybrid hydrogels were assessed by SEM. SEM images revealed a microporous structure in all three conditions (Figure 2B). However, the SEM image of the HA-Tyr hydrogel showed fewer pores compared to G-Try, which has a dense microstructure. As expected, the HA-Tyr/G-Tyr hybrid hydrogel displayed a unique structure combining the features of HA-Tyr and G-Tyr that resulted in both dense and porous microstructures. The dense structure of HA-Tyr/G-Tyr (2:1) was seen to turn into a more porous structure when the ratio of G-Tyr was increased (1:1 and 1:2). This transition in porosity was also confirmed by a liquid displacement test, which revealed the percentage porosity of HA-Tyr/G-Tyr at 2:1, 1:1, and 1:2 (v/v) to be 42.1%, 58.9%, and 83.0%, respectively (*n* = 3) (Figure S9).

### 3.3 Cell Viability and Proliferation on HA-Tyr/G-Tyr Components and Hybrid Gels

**Figure 3:**
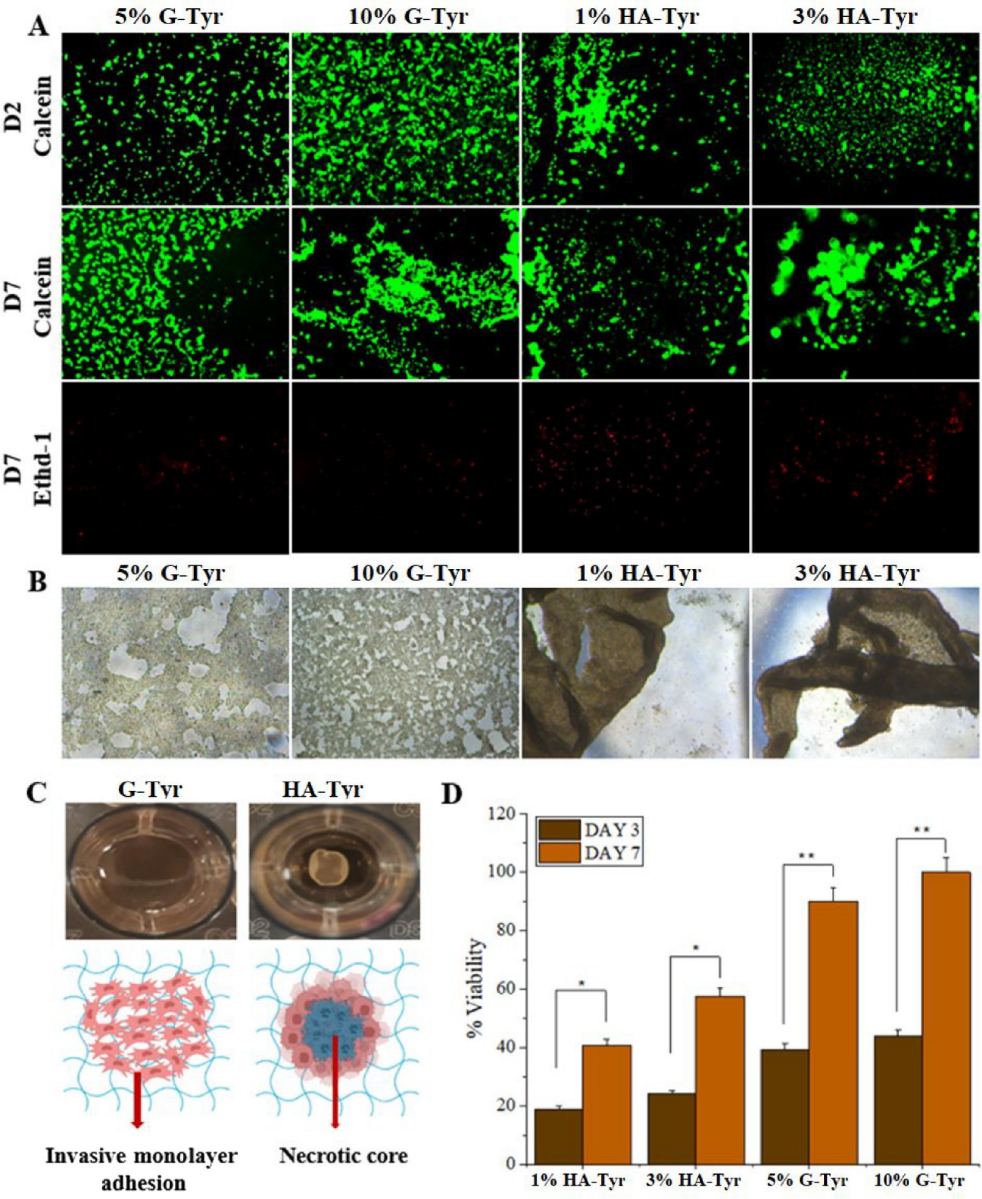
*In vitro* cytotoxicity of gel components. (A) Cell viability assay for HT-29 cells that were cultured on G-Tyr (5 and 10% wt) and HA-Tyr (1 and 3% wt) hydrogels for 2 and 7 days. (B) Microscopic images of HT-29 cells that were cultured on HA-Tyr and G-Tyr hydrogels. (C) Macroscopic images and schematic representation of HT-29 cells on HA-Tyr and G-Tyr hydrogels. (D) Cell proliferation (XTT) test on G-Tyr and HA-Tyr hydrogels (*p < 0.05, **p < 0.005).

We assessed the proliferation and viability of HT-29 cells (40 000 cells/gel) on the synthesized G-Tyr hydrogels (5% and 10% wt), HA-Tyr hydrogels (1% and 3% wt), as well as on the hybrid hydrogels of HA-Tyr/G-Tyr at 2:1, 1:1, 1:2 volumetric ratios using calcein-AM and EthD-1 staining. Cells cultured on both HA-Tyr and G-Tyr showed good viability after 7 days, however, more death cells were seen in HA-Tyr hydrogels compared to G-Tyr **(**Figure 3A). In addition, we observed cell clustering on HA-Tyr hydrogels due to the lack of cell adhesive motifs whereas cells spreading was observed on G-Tyr hydrogels (Figure 3B,C), due to its inherent ability to present cell binding epitopes. Regarding the hybrid hydrogels, the cells showed a good viability in all the combinations; however, the cells exhibited a more aggregated morphology on HA-Tyr/G-Tyr (2:1, v/v) compared to HA-Tyr/G-Tyr (1:2, v/v) (Figure S10). Furthermore, we carried out XTT test to validate the cell viability and proliferation assays. Figure 3D showed that the cells maintained their mitochondrial activities on all the hydrogels and HT-29 cells proliferation increased significantly from day 3 to 7 on both HA-Tyr and G-Tyr hydrogels, albeit, G-Tyr hydrogels favored more cell proliferation than the HA-Tyr hydrogels. Put together, G-Tyr hydrogels promote HT-29 cell proliferation possibly due to the intrinsic cell adhesive property of G-Tyr. On the other hand, HA-Tyr hydrogels favor cell clusters formation due to the lack of intrinsic adhesive epitope. Such cluster formation can lead to unfavorable metabolic stress due to an insufficient nutrient and oxygen supply.^35^ This observation was confirmed by XTT testing on hybrid hydrogels, which showed slightly higher cell proliferation at HA-Tyr/G-Tyr (1:2, v/v) compared to HA-Tyr/G-Tyr (2:1, v/v).

### 3.4 Assessment of HT-29 Cell Adhesion on HA-Tyr/G-Tyr Two-Component Hydrogels

**Figure 4:**
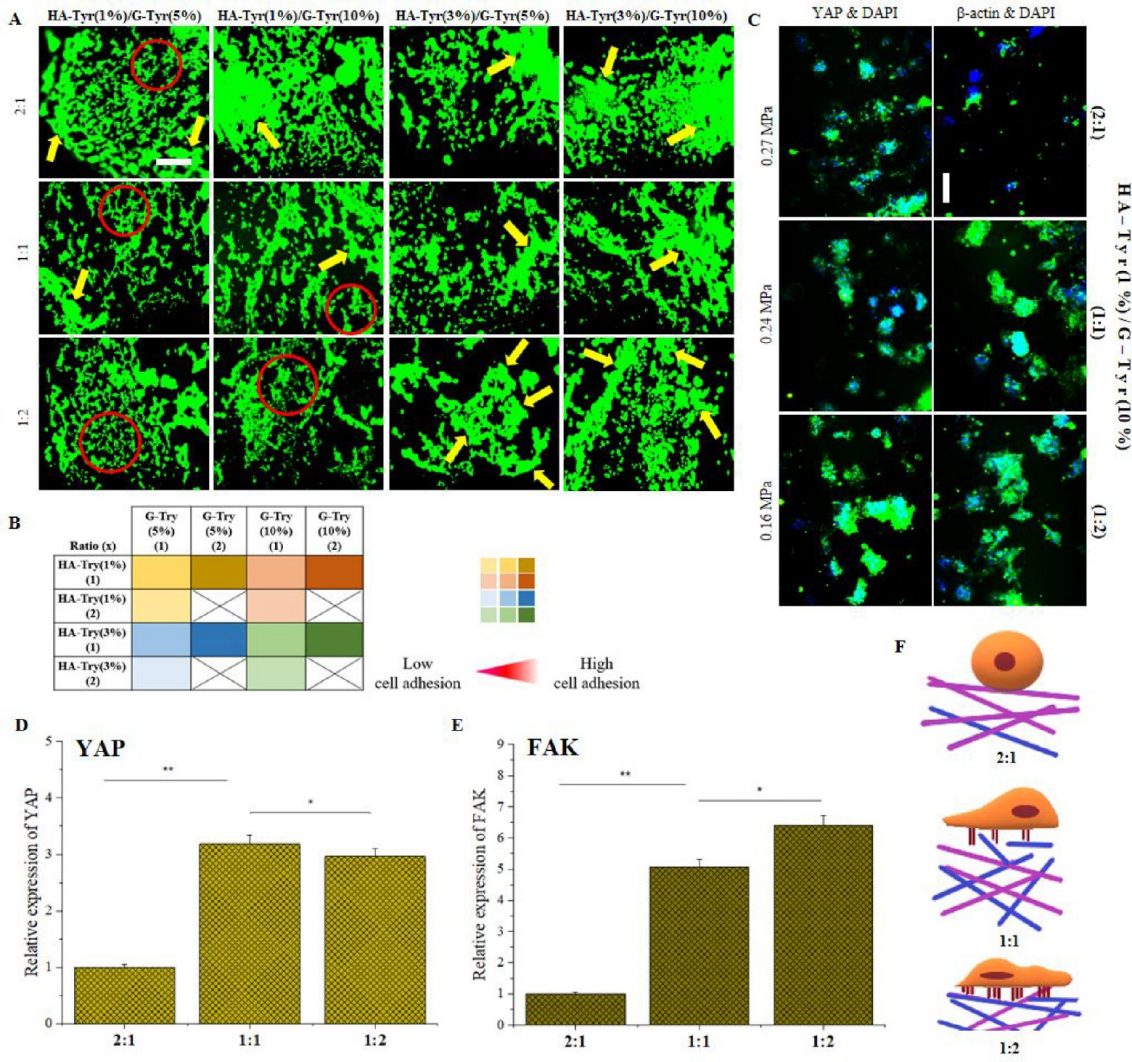
HA-Tyr/G-Tyr is able to control cancer cell adhesion/clustering in stiffness-independent manner. Controlling cell adhesion and clustering. (A) Assessment of cell adhesion and clustering through calcein-AM staining after 5 days of culture. Scale bar 200 μm. (B) Table representing the combinations of HA-Tyr and G-Tyr that were used to assess cell behavior. (C) Immunostaining for β-actin and YAP on HA-Tyr/G-Tyr hydrogels (1%–10% wt) with varying component ratio (2:1, 1:1, 1:2 v/v). Cell nuclei were stained with DAPI. Scale bar: 200 μm. (D,E) Gene expressions for YAP and FAK, two mechanotransduction genes. Statistical significance was determined using a one-way ANOVA followed by Tukey’s post hoc test (*n* = 3, technical replicate = 3) (*p > 0.05, **p < 0.05). (F) Schematic illustration of the effect of component ratio on cell adhesion and mechanosensation.

We carried out calcein-AM staining on the HT-29 cells to determine the optimal two-component hydrogel formulation with tunable property to control monolayer or cluster forming ability of HT-29 cells. Such a platform will also provide the opportunity to control metastatic behavior of cancer cells, particularly along the tissue basal membrane. We prepared various hybrid hydrogels of HA-Tyr (1% and 3% wt) and G-Tyr (5% and 10% wt) by mixing aqueous solutions of the gelators at 1:1, 1:2, and 2:1 volumetric ratio. The hybrid hydrogels prepared at 2:1 v/v showed low cell adhesion but promoted formation of irregular cell clusters as expected and reported elsewhere^36^ (Figure 4A). The 3D cell clusters (yellow arrows) are reminiscent of *in vivo* tumor morphogenesis.^37^ In contrast, cells cultured on hybrid hydrogels composed of 1:1 ratio of HA-Tyr (1% wt) and G-Try (10% wt) displayed a monolayer organization with less cluster cells, whereas cells seeded on hydrogels composed of 1:2 ratios of HA-Tyr and G-Tyr promoted the formation of a cell monolayer with little or no clustered cells formation (Figure 4A). In all the formulation regimes, hybrid hydrogels prepared with HA-Tyr(1%)/G-Tyr(5%) and HA-Tyr(1%)/G-Tyr(10%) promote the formation of cell monolayers. On the other hand, hydrogels prepared with HA-Tyr(3%)/G-Tyr(10%) predominantly drive cluster formation, whereas HA-Tyr(3%)/G-Tyr(5%) hydrogels favor the formation of both cell monolayer and clusters. The combinations of various hybrid hydrogels tested to tune cell adhesion have been summarized in Figure 4B. Put together, both HA-Tyr(1%)/G-Tyr(5%) and HA-Tyr(1%)/G-Tyr(10%) present the opportunity to tune cancer cell adhesion while HA-Tyr facilitates cluster formation in a concentration-dependent manner. Given the obvious indication that HA-Tyr(1%)/G-Tyr(10%) hydrogels can drive the HT-29 cells transition between cluster and monolayer formation, we focused our subsequent studies on this class of hybrid hydrogels.

To further confirm the ability of HA-Tyr/G-Tyr hybrid hydrogels to tune HT-29 cell adhesion, we performed immunofluorescent staining for β-actin and YAP on HA-Tyr(1% wt)/G-Tyr(10% wt) hydrogels prepared at various mixing ratios (2:1, 1:1, 1:2, v/v). Expressions of both β-actin and YAP increased as we changed the gelator mixing ratios from 2:1 to 1:2 (Figure 4C). We reasoned that the observed downregulation in expression of β-actin and YAP might be due to the inability of HA-Tyr/G-Tyr (2:1) to promote cell adhesion, but spheroid-like formation hinders effective cytoskeleton organization (Figure 4C). In contrast, the cells on HA-Tyr/G-Tyr (1:2) feel and sense the matrix more strongly due to the adhesive character of G-Tyr rich hydrogels, which results in an upregulation of the expression of YAP (Figure 4C).

To support our findings with immunostaining assays, we conducted gene expression analysis for two mechanotransduction markers, YAP and focal adhesion kinase (FAK). We found that YAP expression was 3.1- and 3-fold higher in HA-Tyr/G-Tyr (1:1) and HA-Tyr/G-Tyr (1:2), respectively, when compared to HA-Tyr rich hybrid hydrogels (Figure 4D). The difference in YAP expression between HA-Tyr/G-Tyr (1:1) and HA-Tyr/G-Tyr (1:2) was not significant. Similarly, the expression of FAK was seen to increase in the HA-Tyr/G-Tyr (1:1) and HA-Tyr/G-Tyr (1:2) hydrogels by 5- and 6.5-fold, respectively, as compared to the HA-Tyr/G-Tyr (2:1) hydrogel (Figure 4E). Again, the difference among HA-Tyr/G-Tyr (1:1) and HA-Tyr/G-Tyr (1:2) was not significant. Given that YAP/FAK expression increases toward G-Tyr rich hydrogels and inversely correlates with the matrix stiffness of HA-Tyr/G-Tyr, it can be concluded that the higher expression of mechanotransduction markers both in immunostaining and gene expression studies results from the cell-adherent character of G-Tyr rich hydrogels. In light of these findings, we established that cell adhesion and clustering can be controlled by tuning the ratio of HA-Tyr and G-Tyr in HA-Tyr/G-Tyr hybrid hydrogels in a YAP/FAK-independent manner (Figure 4F).

### 3.5 HA-Tyr/G-Tyr Hydrogels Control Cancer Cell Metastasis

**Figure 5:**
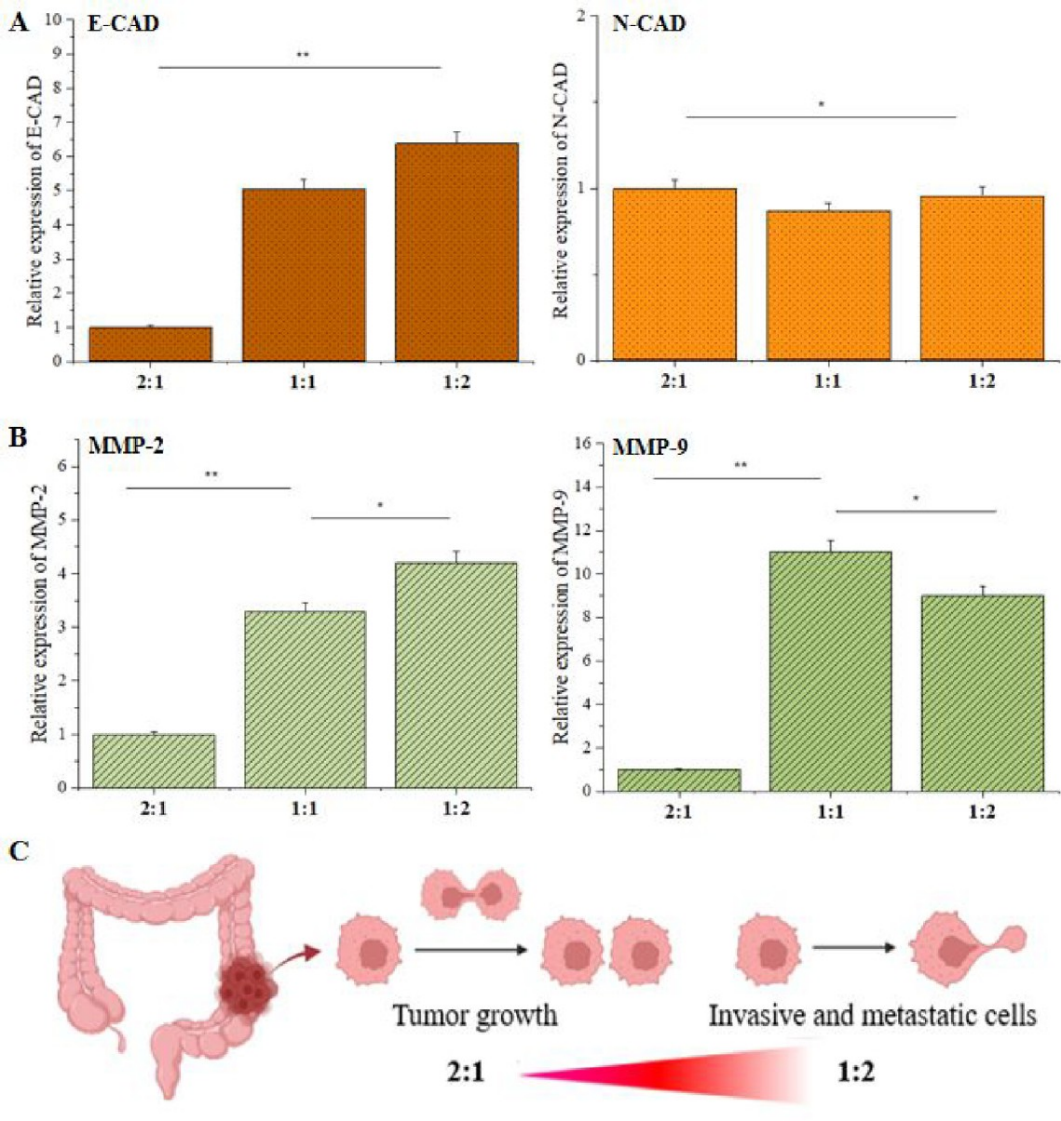
HA-Tyr/G-Tyr-mediated control of cancer cell metastasis. (A) EMT (ECad and NCad) and (B) metastatic (MMP-2 and MMP-9) gene expressions in HT-29 cells that were cultured on HA-Tyr/G-Tyr (1%–10% wt) hybrid hydrogels with different component ratios (2:1, 1:1, 1:2 v/v) for 5 days. Statistical significance was determined using a one-way ANOVA followed by Tukey’s post hoc test (*n* = 3, technical replicate = 3) (*p < 0.05, **p < 0.005). (C) Schematic illustration of working principle of HA-Tyr/G-Tyr hydrogels.

The ultimate goal of this study is to harness the tunable surface chemistry of HA-Tyr/G-Tyr hydrogels to control the metastatic behavior and cell fate of human colorectal adenocarcinoma cells. To do this, we prepared both HA-Tyr rich and G-Tyr rich hybrid hydrogels using 2:1, 1:1, and 1:2 volumetric ratios of HA-Tyr (1% wt) and G-Tyr (10% wt) and cultured HT-29 cells on the hydrogels for 5 days. Then, we assessed the expression of E-cadherin and N-cadherin (EMT markers) as well as MMP-2 and MMP-9 (metastasis markers) on the cells. The cells cultured on the HA-Tyr/G-Tyr (1:1) and HA-Tyr/G-Tyr (1:2) hydrogels, respectively, showed about 10 and 14 times higher expression of E-cadherin (*p* < 0.05) compared to HA-Tyr/G-Tyr (2:1), which implies increased epithelial character in HT-29 cultured on HA-Tyr/G-Tyr (1:2) hydrogels (Figure 5). Interestingly, the differential expression of N-cadherin between various HA-Tyr/G-Tyr hybrid hydrogels was not statistically significant (Figure 5A). This suggests that although the cells assembled into spheroid-like aggregates, they lacked tumor-like mesenchymal character. The underlying reason for the preservation of the epithelial phenotype on HA-Tyr/G-Tyr (1:2) was due to the high cell–matrix interaction, whereas the loss of epithelial phenotype on HA-Tyr/G-Tyr (2:1) is attributed to the formation of clusters, independent of metastasis.^38^

We observed 3.3- and 4.1-fold increases in the expression of MMP-2 on HT-29 cells cultured on HA-Tyr/G-Tyr (1:1) and HA-Tyr/G-Tyr (1:2) hydrogels, respectively, when compared to cells cultured on HA-Tyr/G-Tyr (2:1) (Figure 5B). The expression of MMP-9 followed a similar trend with 11- and 9.2-fold expression levels on HA-Tyr/G-Tyr (1:1) and HA-Tyr/G-Tyr (1:2), respectively. We attribute the significant increase in the expressions levels of MMP-2 and MMP-9 on G-Tyr rich hydrogels to the strong cell–matrix interactions and invasive spreading of cells without cluster formation. This result shows the possibility to modulate the metastatic character of colorectal cancer cells independent of EMT by controlling the surface cell adhesive property presented by the hybrid hydrogels (Figure 5C). This finding is consistent with previous reports by others that metastatic genotype is overexpressed by cancer cells cultured on soft and cell binding matrices.^39^ Moreover, it has been established that expression of E-cadherin plays vital roles in the development of metastasis genotype.^40,41^

### 3.6 HA-Tyr/G-Tyr Hydrogels Control Cancer Cell Death Pathways

**Figure 6:**
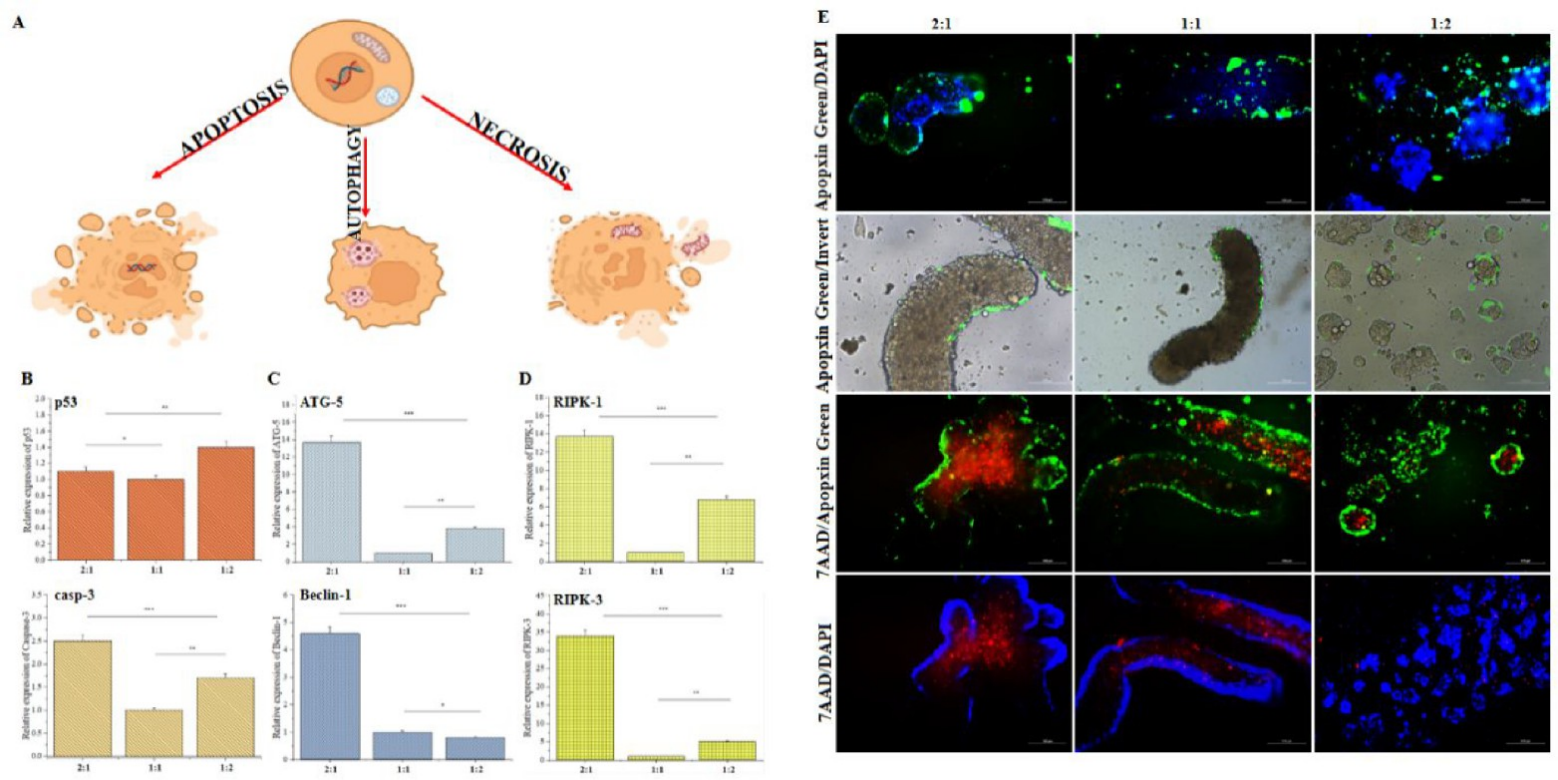
HA-Tyr/G-Tyr-mediated activation of cell death pathways. (A) Strategy followed in order to reveal the HA-Tyr/G-Tyr-instructed death pathways. Gene expressions of (B) apoptosis markers (Casp-3 and p53), (C) autophagy markers (ATG-5 and Beclin-1), and (C) necrosis markers (RIPK-1 and RIPK-3) in HT-29 cells that were cultured on HA-Tyr/G-Tyr (1%–10% wt) hydrogels at 2:1, 1:1, and 1:2 ratios for 5 days. Statistical significance was determined using one-way ANOVA followed by Tukey’s post hoc test (*n* = 3, technical replicate = 3). (E) Apoptosis (Apopxin, green)/necrosis (7AAD, red) assay for HT-29 cells that were cultured on HA-Tyr/G-Tyr (1%–10% wt) hydrogels at 2:1, 1:1, and 1:2 ratios for 5 days. (*p > 0.05, **p < 0.05, ***p < 0.001).

We assessed the ability of HA-Tyr/G-Tyr hybrid hydrogels to instruct HT-29 cell fate by gene expression analysis for several cell death pathways including apoptosis (Casp-3, p53), necrosis (RIPK3, RIPK1) and autophagy (ATG-5, Beclin-1) (Figure 6A). The results showed that apoptosis was suppressed in HT-29 cells by HA-Tyr/G-Tyr hydrogels in all conditions, however, apoptotic pathway dependent on Casp-3, which is involved in the mechanism of oxidative phosphorylation, was found to be 2.5-fold increase in cells that were cultured on HA-Tyr/G-Tyr (2:1) hydrogels compared to HA-Tyr/G-Tyr (1:1) hydrogels (Figure 6B). As expected, autophagic gene expression (ATG-5) was decreased by 14- and 3.5-fold in HT-29 cells when they were cultured on HA-Tyr/G-Tyr (1:1) and HA-Tyr/G-Tyr (1:2) hydrogels compared to HA-Tyr/G-Tyr (2:1) hydrogels, respectively. Beclin-1 was upregulated (∼4.5-fold) in HT-29 cells when they were cultured on HA-Tyr/G-Tyr (2:1) hydrogels (Figure 6C). In the case of necrosis, cells cultured on HA-Tyr/G-Tyr (2:1) hydrogels expressed RIPK-1 and RIPK-3 by 14- and 34-fold higher than HA-Tyr/G-Tyr (1:1) hydrogels. The expression levels of RIPK-1 and RIPK-3 were upregulated by 7- and 5-fold in HT-29 cells when cultured on HA-Tyr/G-Tyr (1:2) and compared to HA-Tyr/G-Tyr (1:1) (Figure 6D). Put together, these findings indicate that HT-29 cells on HA-Tyr/G-Tyr (2:1) are more prone to necrosis than apoptosis, which is also in consistent with the upregulated Casp-3 expression on this hydrogel.

It is known that apoptosis and autophagy, which are necessary for cell regeneration, are suppressed by the cells acquiring a cancer cell genotype and when the cells are involved in the metastatic phase.^42^ The observed decrease in the metastatic genotype and increase in the apoptotic and autophagic genotypes from HA-Tyr/G-Tyr (1:2) to HA-Tyr/G-Tyr (2:1) hydrogels in our study corroborate this fact. On the other hand, there are findings showing that necrosis not only is associated with tumor development but also is activated due to a glucose and oxygen deficiency in large cell clusters and spheroids.^43^ Therefore, it can be concluded that the observed increased level of necrosis in the cells cultured on HA-Tyr/G-Tyr (2:1) is not associated with metastasis in our case, whereas it is predominant in the cluster body.

To corroborate gene expression analyses, we also performed an apoptosis/necrosis assay, that stains apoptotic (Apopxin, green) and necrotic (7AAD, red) cells. Fluorescent microscopy images showed that there appeared only a few apoptotic cells mainly located at the border of large cell clusters and colonies when the cells where cultured atop HA-Tyr/G-Tyr (2:1) (Figure 6E). The level of apoptotic cells increased slightly with increasing concentration of HA-Tyr in the 1:2 to 2:1 hydrogels. Moreover, Apopxin/DAPI double staining images demonstrated the presence of active cancer cells on our hydrogels without undergoing DNA damage as revealed by DAPI localization in the nuclei of the cells. In the case of HA-Tyr/G-Tyr (2:1, 1:1), DAPI was localized within cells located at the border of large cell clusters, while it was observed both at the borders and within the colonies in HA-Tyr/G-Tyr (1:2). We reasoned that these findings can be correlated with the metastasis-inducing capacity of HA-Tyr/G-Tyr (1:2) since cell activity and cell resistance to DNA damage reflect the metastatic genotype of cancer cells.^44,45^ On the other hand, HT-29 cells, when they were cultured on HA-Tyr/G-Tyr (2:1), form a necrotic core (red) due to insufficient cell–matrix interaction and resultant cluster formation (Figure 6E). Such a cluster formation restricts intake of sufficient oxygen and glucose, which can cause necrotic cell death. This observation strongly correlates with the increased gene expression of Casp-3 (a marker correlating oxidative stress and aopotosis) when the cells were cultured on HA-Tyr/G-Tyr (2:1).

### 3.7 HA-Tyr/G-Tyr Hydrogels Reconstruct Metabolomics Structure of HT-29 Cells

**Figure 7:**
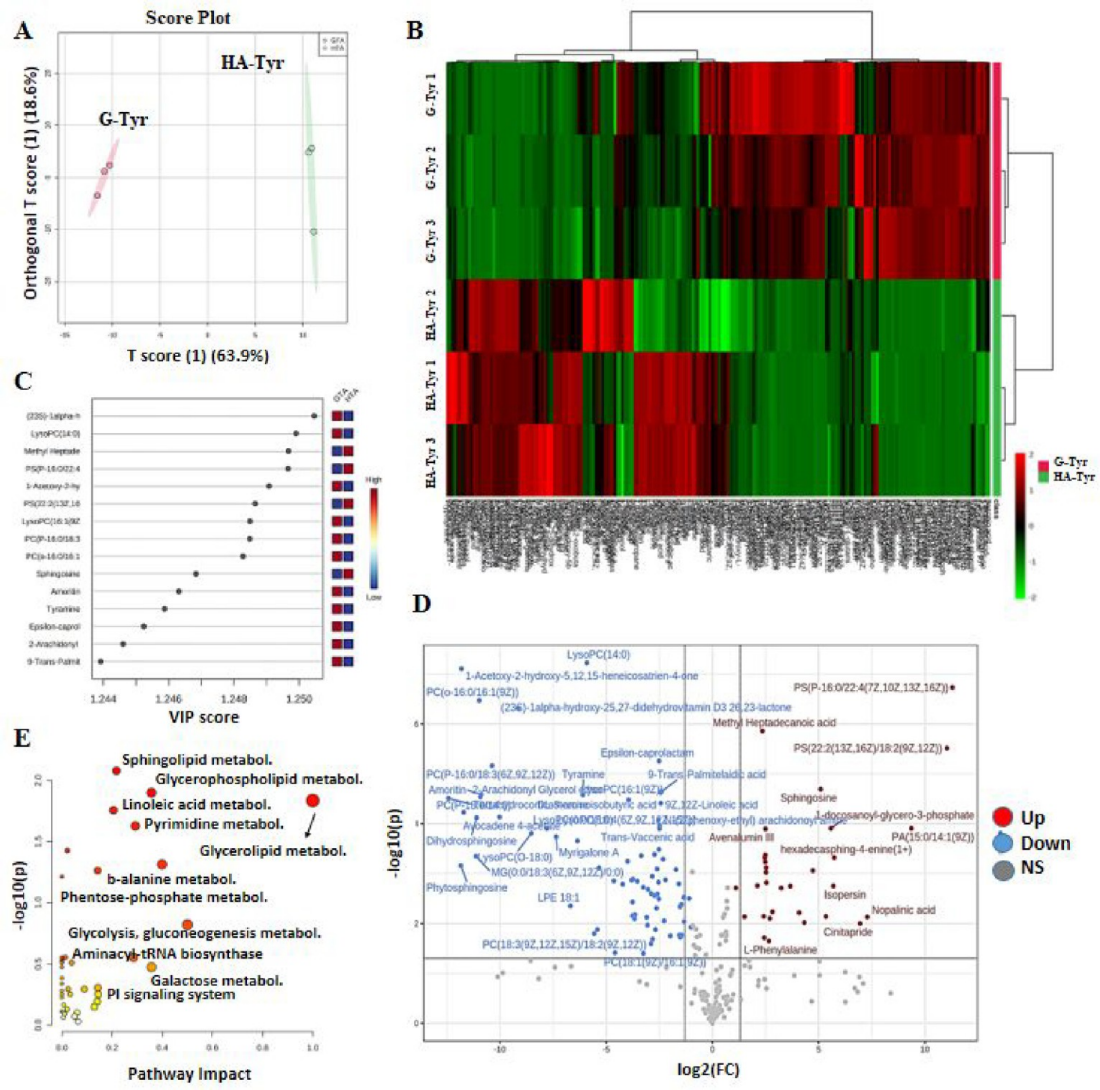
Effects of hybrid hydrogel components on metabolomics discrimination in HT-29. (A) PLS-DA diagram showing the discrimination in metabolite profile in HT-29 cells when they were cultured on HA-Tyr and G-Tyr hydrogels for 5 days. (B) Hierarchical clustering analysis obtained by altered metabolites in HT-29 cells that were cultured on HA-Tyr and G-Tyr hydrogels. (C) VIP score plot listing the top discriminated metabolites among groups. (D) Volcano scatter plot indicating the up/down-regulated metabolites as well as metabolites in similar level among groups. (E) Pathway impact graph representing the most significantly affected pathways in HT-29 cells when they were cultured on HA-Tyr and G-Tyr hydrogels. Data analyses have been conducted with MetaboAnalyst (*n* = 3, technical replicate = 3).

In order to mechanistically elucidate the behaviors of HT-29 cells that we cultured on HA-Tyr/G-Tyr hydrogels, we performed an untargeted metabolomics analysis to interpret the metabolic effect of hybrid gel parameters (HA-Tyr and G-Tyr) on HT-29 cells. A total of 11 422 (positive ion mode) and 8394 (negative ion mode) mass features were detected and 113 metabolites were identified by LC qTOF-MS. Also, 763 metabolites were detected and 106 metabolites were identified by GC-MS (data available in the SI). In order to investigate the differences in the metabolomics profiles of HT-29 cells on HA-Tyr and G-Tyr hydrogels, partial least-squares-discriminant analysis (PLS-DA) was conducted. PLS-DA analysis showed a remarkable discrimination between the metabolic phenotypes of the cells cultured on HA-Tyr and G-Tyr (Figure 7A), validated by *t* test (Figure S11). The metabolomics pattern, obtained by color-coding hierarchical cluster analysis that represents the intensity of the metabolites within groups, was found to be distinct for G-Tyr and HA-Tyr (Figure 7B). The Variable Importance in Projection (VIP) plot was supplied to provide an understanding of the most influential metabolites between groups (Figure 7C). Fifteen metabolites that altered most significantly between HA-Tyr and G-Tyr included lactone, methyl heptadecanoic acid, 1-acetoxy-2-hydroxy-5,12,15-heneicosatrien-4-one, and sphingosine. Strikingly, these metabolites were upregulated only in G-Tyr and downregulated in HA-Tyr. This observation highlights that the metabolites differentiated between two groups at the top level were G-Tyr-derived. In order to examine the metabolomics diversity between G-Tyr and HA-Tyr, we generated a volcano scatter graph that reflects the overall distinction between the two groups (Figure 7D). Lactone, 1-acetoxy-2-hydroxy-5,12,15-heneicosatrien-4-one, and ε-caprolactam were the major metabolites that were significantly downregulated between the two groups, while methyl heptadecanoic acid and sphingosine were upregulated. Finally, sphingolipid metabolism (*p* < 0.008), glycerophospholipid metabolism (*p* < 0.013), linoleic acid metabolism (*p* < 0.015), pyrimidine metabolism (*p* < 0.018), glycerolipid metabolism (*p* < 0.0024), and pantothenate and CoA biosynthesis (*p* < 0.037) were the leading altered pathways having roles in the adhesion of HT-29 cells on HA-Tyr and G-Tyr hydrogels (Figure 7E). Based on the overall variation in metabolites and associated pathways, we conclude that a clear distinction was observed in the metabolite profiles of HT-29 cells cultured on HA-Tyr and G-Tyr hydrogels that will be expected to affect the functional phenotypes level.

**Figure 8:**
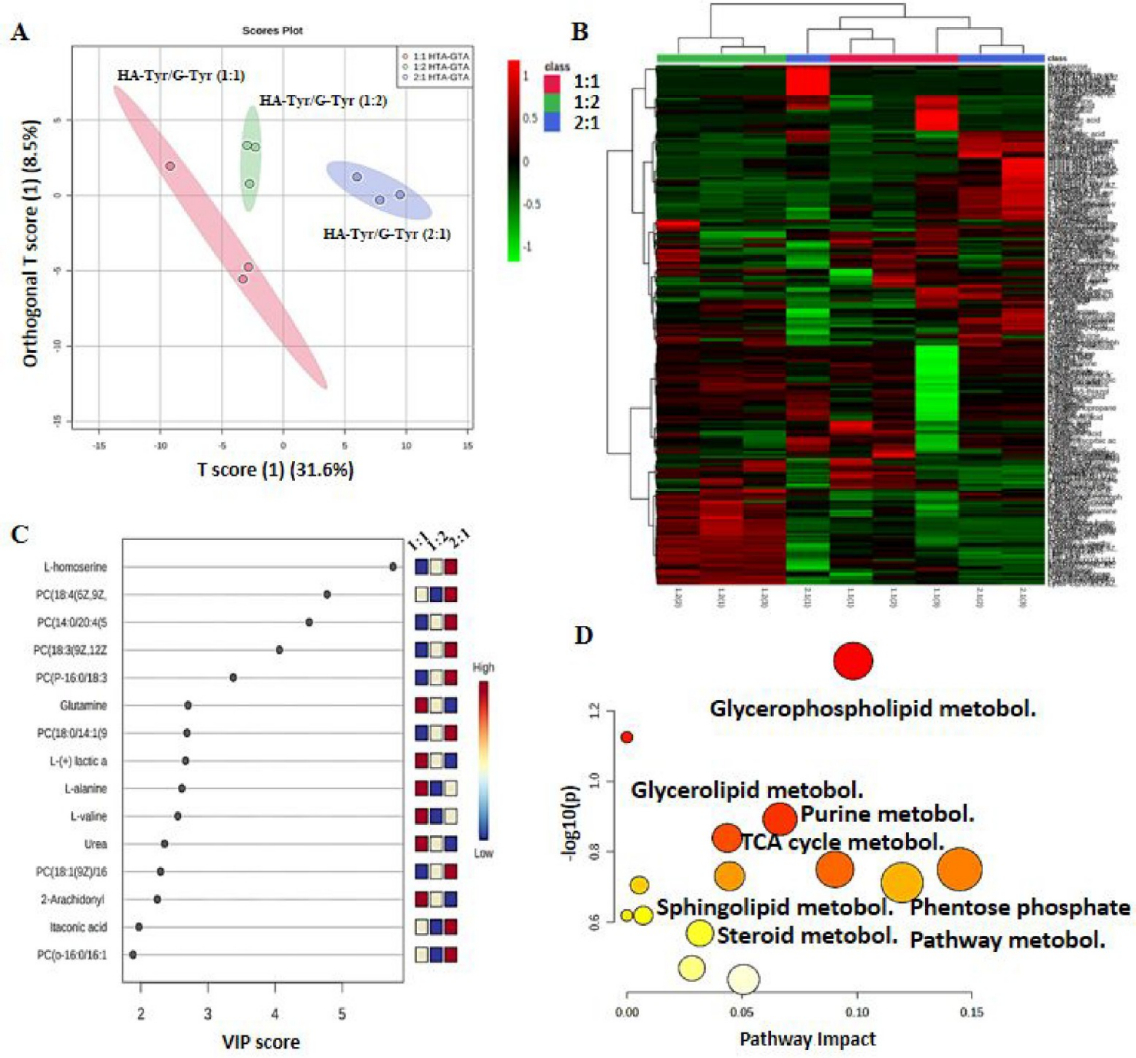
Effect of component ratio on metabolomics structure. (A) PLS-DA diagram showing the discrimination in metabolite profile in HT-29 cells when they were cultured on HA-Tyr/G-Tyr (1%–10% wt) hydrogels with varying component ratios (2:1, 1:1, 1:2 v/v) for 5 days. (B) Hierarchical clustering analysis obtained by altered metabolites in HT-29 cells that were cultured on (1%–10% wt) hydrogels with varying component ratios (2:1, 1:1, 1:2 v/v). (C) VIP score plot listing the top discriminated metabolites among groups. (D) Pathway impact graph representing the most significantly affected pathways in HT-29 cells when they were cultured on HA-Tyr/G-Tyr (1%–10% wt) hydrogels with varying component ratios (2:1, 1:1, 1:2 v/v). Data analyses have been conducted with MetaboAnalyst (*n* = 3, technical replicate = 3).

In order to elucidate the effects of component ratio on metabolomics structure of HT-29 cells, we performed a comprehensive untargeted metabolomics analysis for HT-29 cells that were cultured on HA-Tyr/G-Tyr (1%–10% wt) hydrogels with varying component ratios (2:1, 1:1, 1:2 v/v). The PLS-DA score graph showed a remarkably high metabolomic distinction between the groups which enabled a clear metabolomic cluster separation, and the replicates in each group showed a highly consistent metabolomic structure (Figure 8A). Metabolomic discrimination between the groups was confirmed by the one-way ANOVA test (Figure S12). The alteration tendency of multiple metabolites exhibited a clear distinction for HT-29 cells cultured on HA-Tyr/G-Tyr hybrid hydrogels prepared with 2:1, 1:1, and 1:2 (v/v) ratios of HA-Tyr and G-Tyr (Figure 8B). The metabolites with a higher impact in discriminating the metabolic pattern of HT-29 cells on different ratios of HA-Tyr/G-Tyr are presented in the VIP plot (Figure 8C). The essential 15 metabolites including l-homoserine and glutamine were upregulated only in cells cultured on HA-Tyr/G-Tyr (2:1), while l-(+)-lactic acid, l-alanine, and urea were upregulated only in cells cultured on HA-Tyr/G-Tyr (1:1), which contributed to the separation of metabolite clusters. l-Homoserin and itaconic acid signified in VIP plot was identified only in HT-29 cells cultured on HA-Tyr/G-Tyr (2:1), whereas, l-alanine, l-valine, and urea were only present in the metabolite profile of cells that were cultured on HA-Tyr/G-Tyr (1:1). The metabolites, which differed significantly between the three groups, perform their functions mostly by influencing the glycerophospholipid metabolism pathway (*p* < 0.05) (Figure 8D).

The up/downregulation of various metabolites confirmed the discriminated metabolomic structure of HT-29 cells upon switching the HA-Tyr/G-Tyr ratios in the hybrid hydrogels. Importantly, sphingosine was highly upregulated in HT-29 cells when they were cultured on HA-Tyr rich hydrogels. In addition, it was determined that sphingolipid metabolism was the primary pathway that created the greatest difference between HA-Tyr and G-Tyr rich hydrogels. The effect of the sphingosine-sphingolipid metabolism pathway on cell adhesion has been reported previously.^45^ Sphingolipids are the basic components of the plasma membrane that regulate the membrane dynamics and ensure the clustering of cells.^46^ Over-regulation of the sphingolipid in the cells increases the sphingomyelinase activity and decreases the sphingomyelin (SM) level by converting SM to ceramide.^47^ This mechanism of sphingolipid metabolism causes degradation of integrins, which are the building-blocks of cell adhesion cascades, through reducing the efficiency of the ligand–receptor interaction.^48^ Besides, glycerol-based fatty acids take part in different metabolic pathways to synthesize more complex types of lipids such as diacylglycerides and triacylglycerides. These transformations emerging energy sources regulate the cell-cycle, proliferation, and survival.^49,50^ Overall, the nonadherent character of HA-Tyr rich hydrogels that drives the cells to form quasi-spheroids mainly alters the sphingosine level to affect cell adhesion. Along with lipid metabolism, alteration in amino acid metabolism was also observed in HT-29 cells when they were cultured on HA-Tyr/G-Tyr (2:1 and 1:1) hydrogels. Considering the VIP plot and hierarchical clustering analysis, l-homoserine was upregulated in the HA-Tyr/G-Tyr (2:1) hydrogels and metabolites such as glutamine, l-alanine, and l-valine were observed to be upregulated in HA-Tyr/G-Tyr (1:1) hydrogels and moderately regulated in HA-Tyr/G-Tyr (2:1) hydrogels. On the other hand, the alteration in amino acid origin metabolites when the cells were cultured on HA-Tyr/G-Tyr (1:2) hydrogels was negligible in contrast to the case of HA-Tyr/G-Tyr (1:1 and 1:2) hydrogels.

## 4 Conclusion

We designed hybrid HA-Tyr/G-Tyr hydrogels that enable us to control cancer cell adhesion, metastasis, and associated death pathways. We observed invasive-like adhesions with high cellular connections in hybrid hydrogels with high concentration of G-Tyr, while cells were observed to form 3D quasi-spheroid structures at high concentration of HA-Tyr. Considering the invasive spreading along with cell aggregation during the transition from 2:1 to 1:2 ratios, we determined HA-Tyr/G-Tyr (1%–10% wt) hydrogels as the formulation that allows to control cancer cell adhesion. A comprehensive gene expression study showed that the metastatic genotype was predominant in HA-Tyr/G-Tyr (1:2) hydrogels, in which the cells adhered and moved invasively compared to the cases of HA-Tyr/G-Tyr (2:1) and HA-Tyr/G-Tyr (1:1) hydrogels. It is known that, during tumor development and metastasis, apoptosis is suppressed in cancer cells and metastatic cells tend to undergo necrosis, which is known as the death pathway of cancer cells. In our platform, the results confirmed that apoptosis was suppressed in cancer cells on HA-Tyr/G-Tyr (2:1) hydrogels and necrosis was found to be significantly predominant. This study has demonstrated that HA-Tyr/G-Tyr hybrid hydrogels have dual roles of adjusting cancer cell adhesion and instructing metastasis and cell death. Importantly, the heterodox correlation between matrix stiffness and YAP/TAZ expression resulted from the variation in the cell-adherent character of hybrid hydrogels in this work. Therefore, it can be concluded that the HA-Tyr/G-Tyr system modulates the adhesion/clustering of cancer cells in a YAP/FAK-independent manner. The designed hybrid hydrogel has great potential for the prevention of primary metastasis after onco-surgery, biomaterial-based cancer research, mechanistic investigations, development of treatments, and drug testing.

## Supporting Information Available

Experimental procedures, characterization of gelatin-tyramine, characterization of hybrid hydrogels, additional notes on the experimental procedure of metabolomics (PDF)
