# Supplementary material for: Tuning the Cell-Adhesive Properties of Two-Component Hybrid Hydrogels to Modulate Cancer Cell Behavior, Metastasis, and Death Pathways
Source: Biomacromolecules. 2022 Sep 22;23(10):4254–67. doi: 10.1021/acs.biomac.2c00733 (PMC9554906; doi:10.1021/acs.biomac.2c00733)
Supplement: Supplementary file 1 — bm2c00733_si_001.pdf [file bm2c00733_si_001.pdf]

Tuning the Cell-Adhesive Properties of Two-Component Hybrid Hydrogels to Modulate Cancer Cell Behavior, Metastasis and Death Pathways

Melis Isik[1], Babatunde O. Okesola[2,3], Cemil Can Eylem[4], Engin Kocak[5], Emirhan Nemutlu[4,6], Emel Emregul[1], Matteo D'Este[7] and Burak Derkus[1,8]*

1. Interdisciplinary Research Unit for Advanced Materials (INTRAM), Department of Chemistry, Faculty of Science, Ankara University, Ankara 06560, Turkey
2. Department of Eye and Vision Science, Institute of Life Course and Medical Sciences, Faculty of Medicine, University of Liverpool, Liverpool, L7 8TX, UK
3. School of Life Science, Faculty of Medicine and Health Sciences, University of Nottingham, Nottingham, NG7 2RD, UK
4. Analytical Chemistry Division, Faculty of Pharmacy, Hacettepe University, Ankara 06230, Turkey
5. Division of Analytical Chemistry, Faculty of Gulhane Pharmacy, Health Science University, Ankara 06018, Turkey
6. Bioanalytic and Omics Laboratory, Faculty of Pharmacy, Hacettepe University, Ankara 06230, Turkey
7. AO Research Institute Davos, Clavadelerstrasse 8, Davos Platz 7270, Switzerland
8. Stem Cell Research Lab, Department of Chemistry, Faculty of Science, Ankara University, Ankara 06560, Turkey

*Corresponding Author: bderkus@ankara.edu.tr

Outline

Section 1: Modification of gelatin with tyramine (G-Tyr)

1.1. Synthesis of G-Tyr

1.2. FTIR spectroscopy

1.3. NMR spectroscopy

Section 2: Hydrogelation of G-Tyr and characterization of G-Tyr hydrogel

2.1. Hydrogelation of G-Tyr

2.2. Scanning electron microscopy (SEM)

2.3. Degredation test

2.4. Equilibrium water intake (swelling) analysis

2.5. Optical transparancy measurements

Section 3: Chemical characterization of two-component hydrogels

Section 4: Porosity of hybrid hydrogels

Section 5: Live-dead assay and proliferation test for hybrid hydrogels

Section 6: Gene expression study

6.1. Primer sequences related to mechanotransduction, EMT, and metastasis genes

6.2. Primer sequences related to apoptosis, autophagy, and necrosis genes

Section 7: Metabolomics analysis

7.1. Sample preparation for metabolomics

7.2. Metabolomics analysis

7.3. Data processing, bioinformatics study, and statistical analysis

7.4. One-way ANOVA test and affected pathways in HT-29 cells on HA-Tyr, G-Tyr, and three different ratios of HA-Tyr/G-Tyr hybrid hydrogels

Section 1: Modification of gelatin with tyramine (G-Tyr)

1.1. Synthesis of G-Tyr

Briefly, gelatin type A (2% w/v, Sigma, Germany) was dissolved in morpholinoethanesulfonic acid (MES, 50mM, Sigma-Aldrich, USA) buffer at 60 °C with continuous stirring. Condensation reaction was initiated by introducing EDC (24 mM, Sigma-Aldrich, USA), NHS (10 mM, Sigma-Aldrich, Germany) and tyramine hydrochloride (58 mM, Sigma, Germany) and pH was adjusted to 6.0. Reaction was allowed to proceed for 12 hours, the final solution was diluted with Dulbecco's phosphate-buffered saline (DPBS), and the solution was dialyzed through a dialysis membrane with 12-14 kDa cut-off to remove byproducts and unreacted reagents. After lyophilization, the product was kept at +4 °C until further use.

1.2. FT-IR spectroscopy

G-Tyr was chemically characterized by FT-IR. The spectra of pure gelatin powder and lyophilized G-Tyr were obtained with a Shimadzu IRAffinity-1 model spectrometer between 400-4000 cm^{-1} spectral wavelength, and the region of interest (400-2000 cm^{-1}) was presented in Figure S1. The gelatin backbone was identified according to corresponding bands such as; the peak in the wavelength at 3300 cm^{-1} shows the -OH stretching vibrations of hydrogen-bonded hydroxyl groups on the polymer; peak at 3000-3100 cm^{-1} shows the N-H stretching of amide-A bonds; the peak at 2920-2950 cm^{-1} indicates the asymmetric aliphatic C-H stretching vibrations; the peak at 2850 cm^{-1} belonging to symmetric aliphatic C-H stretching vibrations; sharp peaks at 1600-1630 cm^{-1} and 1500-1535 cm^{-1} indicate the amide-I and amide-II β-sheet structure, respectively. In addition, peaks at 1386 cm^{-1} and 1469 cm^{-1} represent the symmetric and asymmetric bending vibrations of methyl groups on the gelation backbone. On the other hand, same bands were observed in the spectra of G-Tyr. Additionally, distinct signal locating between 3300-3500 cm^{-1} wavelength in G-Tyr confirms the success in conjugation of aromatic phenol unit of tyramine with carboxyl group of gelatin.[1] However, this peak was not observed in unmodified gelatin.

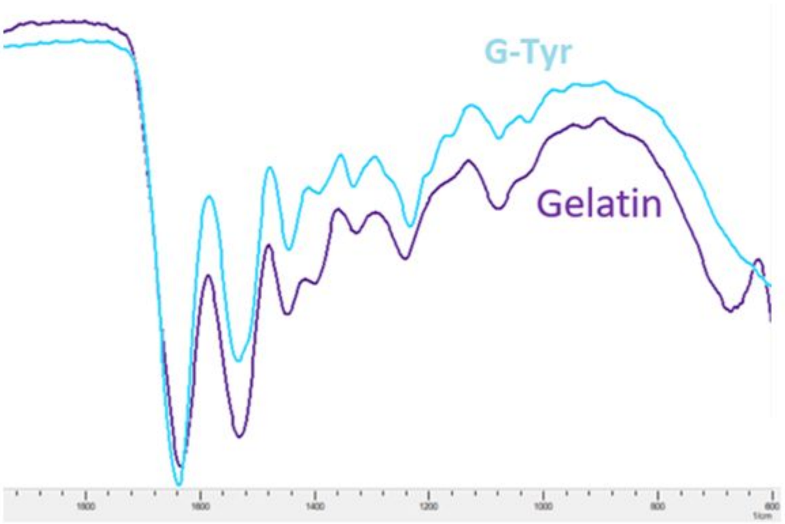

Figure S1. FT-IR spectra depicting the pure gelatin and G-Tyr

1.3. NMR Spectroscopy

G-Tyr was also characterized by nuclear magnetic resonance (NMR) spectroscopy. H^1-NMR spectra related to gelatin and G-Tyr were obtained with a Bruker Biospin spectrometer between 0-10 ppm. H^1-NMR spectroscopy analysis of unmodified gelatin and G-Tyr indicated the common peaks related to gelatin backbone. Unlikely, specific peaks observed in the spectra of G-Tyr at d = 7.2 ppm and d = 6.9 ppm were specific for the phenyl protons of tyramine (Figure S2), which confirms the success of conjugation of tyramine with pure gelatin.[2]

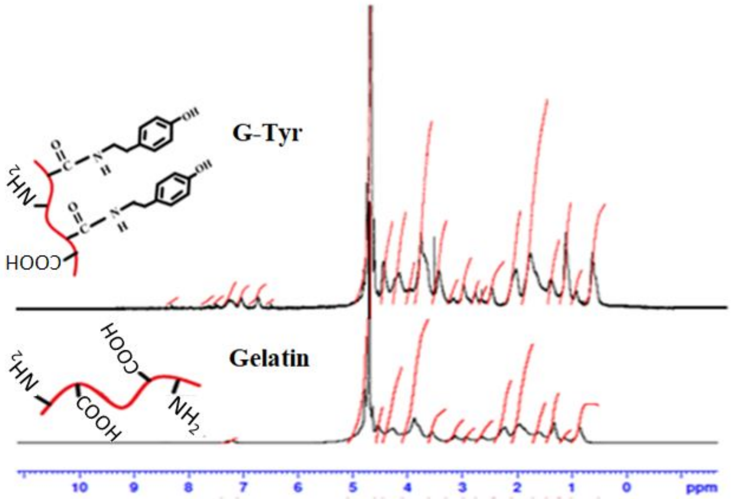

Figure S2. NMR spectra of pure gelatin and G-Tyr

Section 2: Hydrogelation of G-Tyr and characterization of G-Tyr hydrogel

2.1. Hydrogelation of G-Tyr

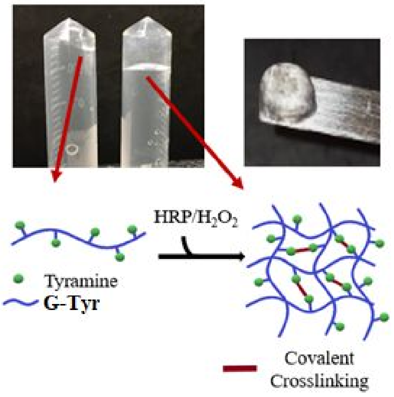

Figure S3 Macroscopic image illustrating the cross-linked and un-crosslinked G-Tyr

G-Tyr (5% and 10% wt.) was dissolved in DPBS untill the homogenity was ensured. HRP (2 U/mL) was added into G-Tyr solution and H_2O_2 (2mM) was added to trigger an orthogonal enzymatic cross-linking resulting in the formation of dityramine bridge.

2.2. Scanning electron microscopy (SEM)

The microstructure of G-Tyr hydrogels (5% and 10% wt.) was investigated under SEM, which exhibited a microporous architecture (Figure S4). As expected, the pore size of G-Tyr (~33 µm in 10% wt.) was found to be lower as compared to G-Tyr (~50 µm in 5% wt.) resulting from a dense matrix structure. This opportunity to tune matrix density enables to control matrix stiffness, thereby, cell behaviors such as adhesion, migration, and invasion will be able to control utilizing the developed hydrogels.[3,4]

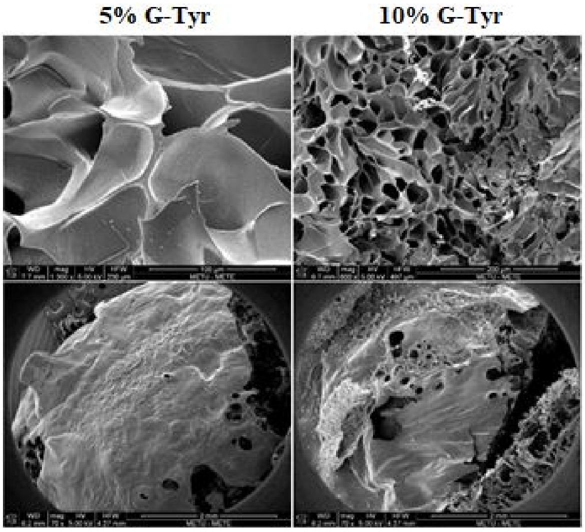

Figure S4. SEM micrograph of G-Tyr hydrogels

2.3. Degredation test

For the degradation test, hydrogels (5% and 10% wt.) were dehydrated overnight by freeze-dryer, weighted (w_o), incubated in DPBS for 1, 2, 5, and 7 day, and the lyophilized hydrogels were weighted to record the final mass (w_f). The degradation rate was calculated using the equation below:

$$\text{Degradation Rate (\%)} = (w_0 - w_f)/w_0 \times 100\%$$

Degradation test of G-Tyr hydrogels (5% and 10% wt.) revealed that G-Tyr (5% wt.) maintained its susceptibility to degradation up to 7 days (Figure S5), while G-Tyr (10% wt.) preserved about 70% of its weight after 7-days incubation. After 7-days of degradation period, the hydrogels kept their integrity with 0.4% weight loss everyday. The gels were observed to preserve their integrity for 77 days (approximately 2.5 months) without being completely degraded. It should also be noted that incubation time of cell culture study in this work was 2-days, and G-Tyr in both 5% and 10% wt. preserve its weight (>75%) after 2-days of incubation.

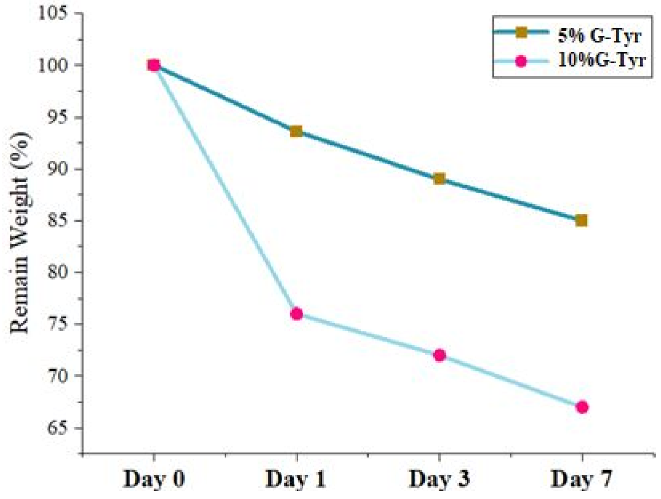

Figure S5. Degredation test of 5% and 10% G-Tyr hydrogels

2.4. Equilibrium water intake (swelling) analysis

For the swelling test, the freeze-dried hydrogels were weighted (w_1), incubated in deionized water for 3 hours to allow complete water intake, and the hydrogels were weighted again in swollen state (w_2). The water intake capacity was evaluated using the following equation:

$$\text{Water intake (\%)} = (w_2 - w_1/w_2) \times 100$$

Equilibrium water intake is an important feature of hydrogels to get knowledge about their water affinity, permeability and diffusion capacity which are important characteristics for the

application of hydrogels.[5] Swelling percentages of hydrogels were yielded as 89.53% (5% G-Tyr) and 90.23% (10% G-Tyr), respectively (Figure S6). There was no significant difference between the swelling characteristics of 5% and 10% G-Tyr hydrogels.

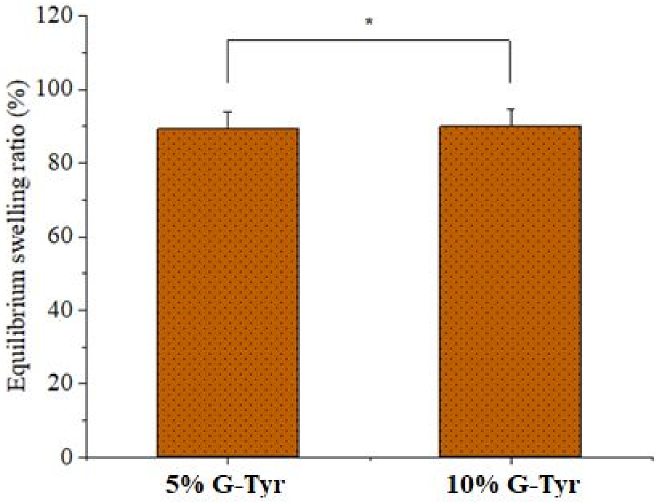

Figure S6. Equilibrium water intake (swelling) graphs of 5% and 10% G-Tyr hydrogel (*p>0.05)

2.5. Optical transparancy measurements

To determine the transparency of G-Tyr hydrogels, hydrogels (5% and 10% wt.) were placed into deionized water containing cuvette and UV absorbance was recorded between 200-700 nm wavelength. Results demonstrated that both hydrogels absorbed the light in UV-region with the wavelenght ranges between 200-350 nm; whereas, G-Tyr hydrogels transmitted light in visible region beyond 350 nm (Figure S7). This shows that light absorbtion in the visible region was low that almost no light was absorbed, while transmittance was high. Obtained results proved the transparancy of G-Tyr (5% and 10% wt.) hydrogels.

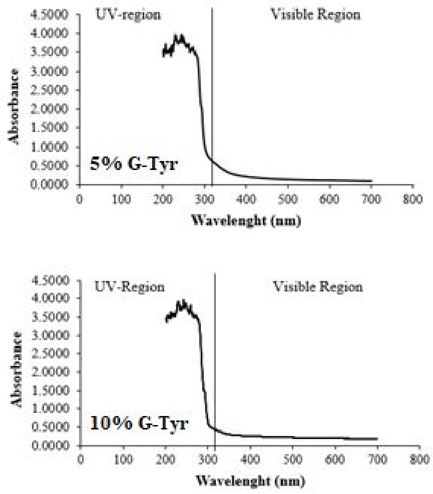

Figure S7. Optical transparancy of G-Tyr hydrogels

Section 3: Chemical characterization of two-component hydrogels

For the structural characterization of HA-Tyr/G-Tyr hybrid hydrogel, we performed ATR-FTIR analysis for HA-Tyr, G-Tyr, and HA-Tyr/G-Tyr. The peaks between 1650 cm^{-1} and 1540 cm^{-1} are attributed to amide I and amide II bonds resulting from the N-acetyl group per repeating unit of HA as well as to the bond between HA and Tyr (Figure S8). The more intense amide peaks seen in G-Tyr was attributed to the polypeptide nature of G-Tyr in addition to the peak indicating the bond between gelatin and Tyr. The peaks at 1250 cm^{-1} in G-Tyr and 1050 cm^{-1} in HA-Tyr represents specific amide III and carbohydrate backbone, respectively. In the case of HA-Tyr/G-Tyr hybrid gel, all characteristic peaks of HA-Tyr and G-Tyr were observed as expected, confirming the structure of the hybrid HA-Tyr/G-Tyr hydrogel.

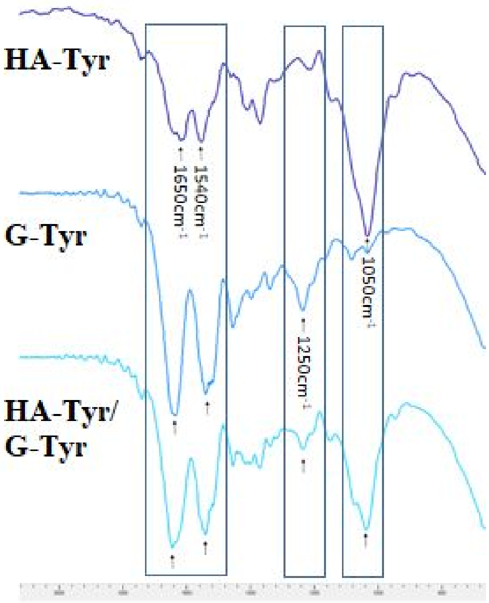

Figure S8. FT-IR specra related to two-component hydrogel and its components

Section 4: Porosity of hybrid hydrogels

Briefly, freeze-dried samples were immersed in a volume (V1) of liquid hexane in an Eppendorf. The total volume of the tube (hexane+hexane-impregnated sample) was considered V2. The volume of the tube when hexane-impregnated sample is removed was noted as V3. The porosity (ε) was calculated utilizing the *equation 1* and given in Table 1.

$$\varepsilon (\%) = [(V1-V3) / (V2-V3)] \times 100 \qquad \textit{equation 1}$$

Table S1 Liquid displacement test results of hybrid hydrogels (2:1, 1:1, 1:2, v/v)

**Volumetric Ratio of Gels**	**V1[uL]**	**V2[uL]**	**V3[uL]**	**%[V1-V3]/[V2-V3]*100**
**2:1 (n=1)**	900	1100	750	**42.9%**
**2:1 (n=2)**	900	1050	750	**50.0%**
**2:1 (n=3)**	900	1200	750	**33.3%**
**1:1 (n=1)**	900	1000	750	**60.0%**
**1:1 (n=2)**	900	950	850	**50.0%**
**1:1 (n=3)**	900	1000	700	**66.7%**
**1:2 (n=1)**	900	950	600	**85.7%**
**1:2 (n=2)**	900	950	700	**80.0%**
**1:2 (n=3)**	900	950	650	**83.3%**

The calculated porosity levels were presented in Figure S9.

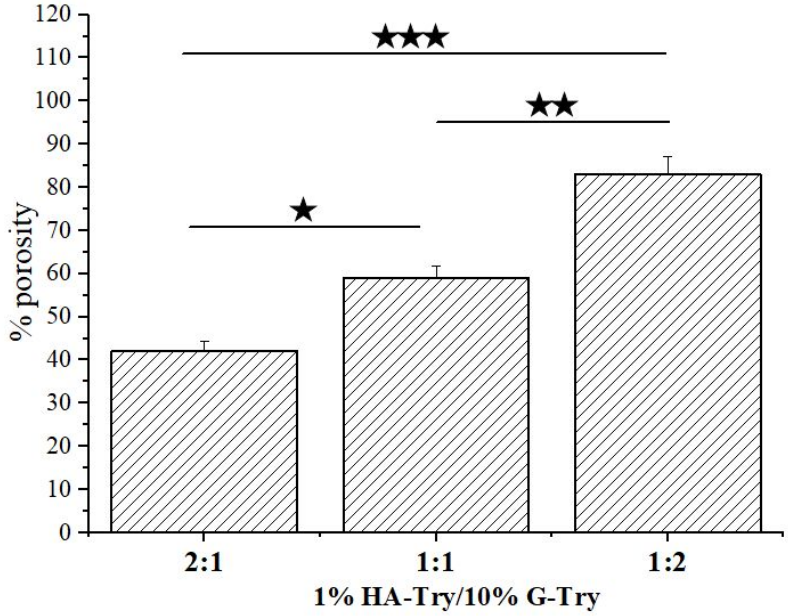

Figure S9. Porosity of hybrid hydrogels (*p<0.05, **p<0.01, ***p<0.001)

Section 5: Live-dead assay and proliferation test for hybrid hydrogels

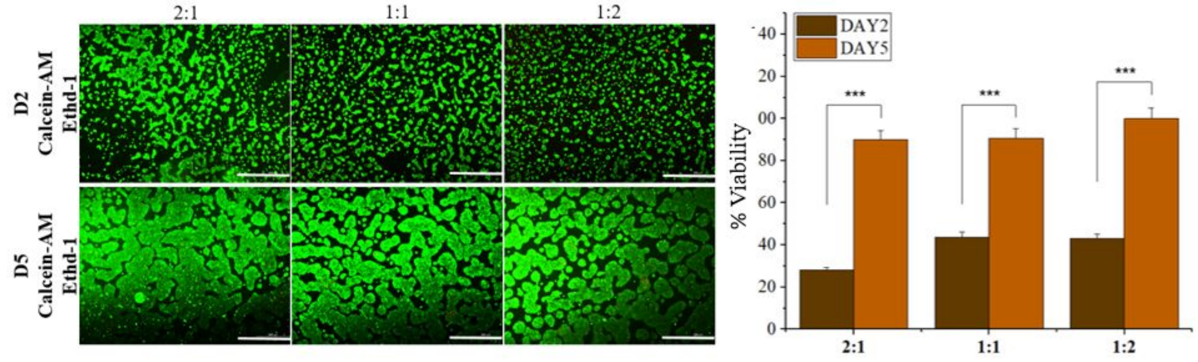

Figure S10. Cytotoxicity of hybrid hydrogels. (A) Calcein-AM (green) and Ethd-1 (red) staining for HA-Tyr/G-Tyr at 2:1, 1:1, 1:2 volumetric ratios (scale bar 200 μm). (B) XTT test for HA-Tyr/G-Tyr at 2:1, 1:1, 1:2 volumetric ratios (***p<0.001).

Section 6: Gene expression study

The list of primers used in gene expression studies can be found in Table S2 and 3.

Table S2 Primer sequences related to mechanotransduction, EMT, and metastasis genes

Genes (Human)	***Primer Sequence (5'-3')***
MMP2	*F*: AGCGAGTGGATGCCGCCTTTAA<br>*R:* CATTCCAGGCATCTGCGATGAG
MMP9	*F:* GCCACTACTGTGCCTTTGAGTC<br>*R*: CCCTCAGAGAATCGCCAGTACT
E-cadherin	*F:* GCCTCCTGAAAAGAGAGTGGAAG<br>*R:* TGGCAGTGTCTCTCCAAATCCG
N-cadherin	*F:* CCTCCAGAGTTTACTGCCATGAC<br>*R:* GTAGGATCTCCGCCACTGATTC
FAK	*F:* GAGATTGAGATGGCACAGAAGC<br>*R:* TGAGCAGCAGTCAGCATTTG
YAP	*F:* CCTCGTTTTGCCATGAACCAG<br>*R:* GTTCTTGCTGTTTCAGCCGCAG
GAPDH (housekeeping gene)	*F:* GAACGGGAAGCTTGTCATCAA<br>*R:* ATCGCCCCACTTGATTTT GG

Table S3 Primer sequences related to apoptosis, autophagy, and necrosis genes

Genes (human)	***Primer Sequence (5'-3')***
P53	*F*: GAGGTTGGCTCTGACTGTACC<br>*R:* TCCGTCCCAGTAGATTACCAC
Caspase-3	*F:* CATGGAAGCGAATCAATGGAC<br>*R*: CTGTACCAGACCGAGATGTCA
ATG-5	*F:* GCAGATGGACAGTTGCACACAC<br>*R:* GAGGTGTTTCCAACATTGGCTCA
Beclin-1	*F:* ACCGTGTCACCATCCAGGAA<br>*R:* GAAGCTGTTGGCACTTTCTGT-3'
RIPK-1	*F:* CGTGCTGAAAGCCGAGATGA<br>*R:* CTTAAAGGAGGCAAGGCCGA
RIPK-3	*F:* GCTACGATGTGGCGGTCAAGAT<br>*R:* TTGGTCCCAGTTCACCTTCTCG

Section 7: Metabolomics analysis

7.1. Sample preparation for metabolomics

Metabolomics analysis was performed with the optimal hybrid gel (HA-Tyr-G-Tyr (1-10% wt.), 1:1, 1:2, 2:1 v/v) to specify the metabolites that alter in response to morphogenesis. To distinguish the metabolite profiles in cancer cells cultured on less and more adhesive matrices, 1% HA-Tyr and 10% G-Tyr were used as controls in this study. To this aim, HT-29 cells (500.000 cell/gel) were seeded on hydrogels in 48-wells and the cell cultures were maintained for up to 5 days. At the end of the culture period, hydrogel-cell constructs were treated with myristic acid (1 ppm) containing methanol-H_2O (1:9 v/v) solution. The plate was soaked into liquid nitrogen for 15 s for the complete disintegration of metabolites and proteins.

7.2. Metabolomics analysis

Metabolomic analysis was performed similarly to that previously described in the literature.[6] Polar metabolites in the upper phase from the samples kept for metabolomic analysis at -80 °C were transferred to new eppendorf tubes and completely dried with a vacuum concentrator to prevent metabolite degradation. The dried samples were methoxylated using a solution of methoxyamine hydrochloride in pyridine and derivatized using N-methyl-N-trimethylsilyl

trifluoroacetamide (MSTFA) and 1% trimethylchlorosilane (TMCS) reagents for 30 minutes at 37°C. Samples were transferred to silanized bottles for analysis and analyzes were performed with Gas Chromatography-Mass Spectrometer (GC-MS) instrument using a DB5-MS column. After the obtained complex chromatograms were separated, the retention times of the peaks were corrected using the SpectConnect software and data matrices were created. Obtained metabolite peaks were illuminated using retention indexed libraries (Fiehn and Golm Database libraries).

7.3. Data processing, bioinformatics study, and statistical analysis

Metabolomic data deconvolution was performed using AMDIS (Automatic Mass Spectral Deconvolution and Identification System) and SpectConnect was used to generate data matrices of metabolite peaks. Metabolomic data matrices were loaded into MetaboAnalyst4.0 and normalized with sum area-mean centering. Any metabolite feature with more than 50% of missing values was excluded from the data matrix. The final data matrix was imported into SIMCA-P+ (v13.0, Umetrics, Sweden) software for multivariate analysis. Principal components analysis (PCA) and Partial Least Squares-Discrimination Analysis (PLS-DA) were performed to reveal class discrimination. The variable importance in projection (VIP) values were used to distinguish the desired metabolites. Pathway analyzes for metabolomics were performed with the metabolites obtained by using online tools and it was determined which pathways were effective in cancer cell behavior with different matrix stiffness.

7.4. One-way ANOVA test and affected pathways in HT-29 cells on HA-Tyr, G-Tyr, and three different ratios of HA-Tyr/G-Tyr hybrid hydrogels

The obtained metabolome data was statistically processed by t-testing and ANOVA test (Figure S10 and 11).

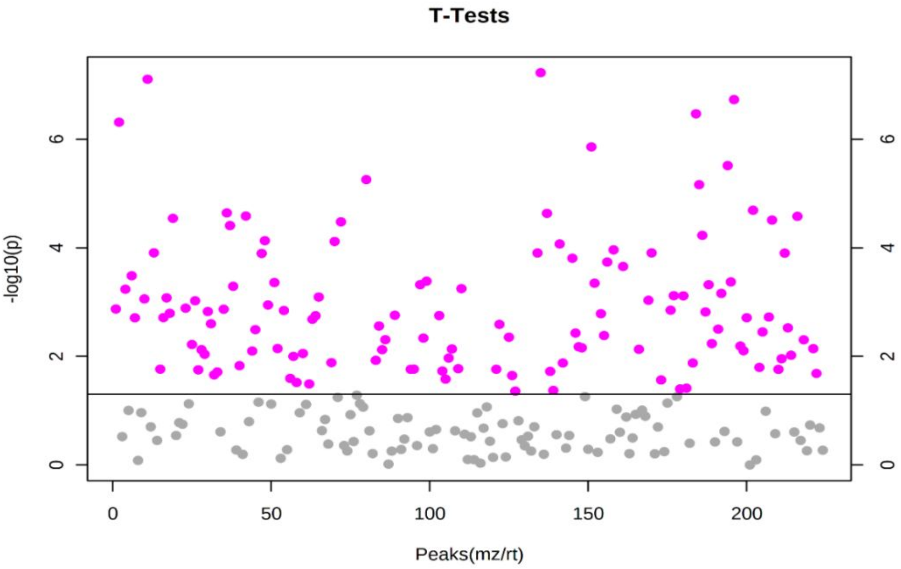

Figure S11. t-test applied for HT-29 cells that were cultured atop HA-Tyr and G-Tyr hydrogels

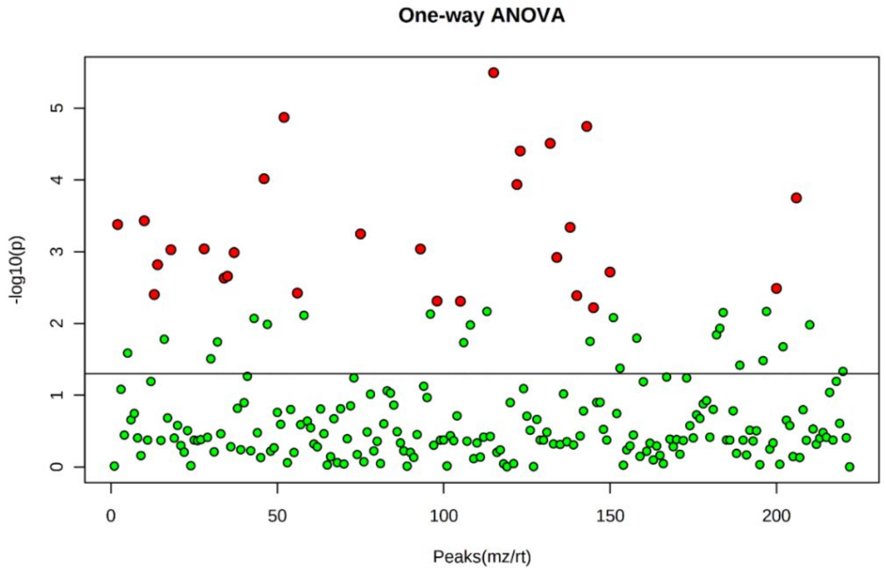

Figure S12. One-way ANOVA test applied for HT-29 cells that were cultured atop 2:1, 1:1, and 1:2 ratios of HA-Tyr/G-Tyr hybrid hydrogels

The pathways affected by up/down-regulated metabolites were presented in Table S4 and S5.

Table S4 Affected pathways in HT-29 cells that were cultured atop HA-Tyr and G-Tyr hydrogels

Pathway Name	p-value	-log10(p)
Sphingolipid metabolism	8.33E-03	2.08E+00
Glycerophospholipid metabolism	1.26E-02	1.90E+00
Linoleic acid metabolism	1.46E-02	1.84E+00
Pyrimidine metabolism	1.75E-02	1.76E+00
Glycerolipid metabolism	2.35E-02	1.63E+00
Pantothenate and CoA biosynthesis	3.74E-02	1.43E+00
beta-Alanine metabolism	4.85E-02	1.31E+00
Pentose phosphate pathway	5.46E-02	1.26E+00
Propanoate metabolism	6.10E-02	1.21E+00
Phenylalanine, tyrosine and tryptophan biosynthesis	1.51E-01	8.22E-01

Table S5 Affected pathways in HT-29 cells comprising the culture onto 2:1, 1:1, and 1:2 ratios of HA-Tyr/G-Tyr hybrid hydrogels

Pathway Name	p-value	-log10(p)
Glycerophospholipid metabolism	4.56E-02	1.34E+00
Ascorbate and aldarate metabolism	7.50E-02	1.12E+00
Purine metabolism	1.28E-01	8.92E-01
Glycerolipid metabolism	1.45E-01	8.39E-01
Citrate cycle (TCA cycle)	1.78E-01	7.50E-01
Ether lipid metabolism	1.78E-01	7.50E-01
Sphingolipid metabolism	1.86E-01	7.31E-01
Pentose phosphate pathway	1.94E-01	7.13E-01
Steroid hormone biosynthesis	1.97E-01	7.06E-01

REFERENCES

1. Patarroyo, J. L., Florez-Rojas, J. S., Pradilla, D., Valderrama-Rincón, J. D., Cruz, J. C., Reyes, L. H. (2020), "Formulation and characterization of gelatin-based hydrogels for the encapsulation of kluyveromyces lactis-applications in packed-bed reactors and probiotics delivery in humans" *Polymers (Basel)*, V:12(6), DOI: https://doi.org/10.3390/polym12061287

2. Ji, C., Khademhosseini, A., Dehghani, F. 2011. Enhancing cell penetration and proliferation in chitosan hydrogels for tissue engineering applications. *Biomaterials*, V:32(36), P:9719-9729, doi: 10.1016/j.biomaterials.2011.09.003.

3. AnilKumar, S., Allen, S. C., Tasnim, N., Akter, T., Park, S., Kumar, A., Chattopadhyay, M., Ito, Y., Suggs, L. J., Joddar, B. (2019), "The applicability of furfuryl-gelatin as a novel bioink for tissue engineering applications" *J. Biomed. Mater. Res. B Appl. Biomater.*, V:107(2), P:314-323, doi: 10.1002/jbm.b.34123.

4. Selyanin, M., Boykov, P., Khabarov, V., Polyak, F. (2015), “Molecular and Supramolecular Structure of Hyaluronic Acid” *Hyaluronic Acid: Preparation, Properties, Application in Biology and Medicine*, P:97-119, DOI:10.1002/9781118695920.ch4

5. Karoyo A.H. & Wilson L.D. (2021), “A Review on the Design and Hydration Properties of Natural Polymer-Based Hydrogels” *Materials,* V:14(5), DOI: https://doi.org/10.3390/ma14051095

6. Dunn, D.W., Broadhurst, D., Begley, P., Zelena, E., Francis-McIntyre, S., Anderson, N., Brown, M., Knowles, J. D., Halsall, A., Haselden, J. N., Nicholls, A. W., Wilson, I. D., Kell, D. B., Goodacre, R. 2011. “Procedures for large-scale metabolic profiling of serum and plasma using gas chromatography and liquid chromatography coupled to mass spectrometry” Nature Protocols, 6; 1060-1083.
